# TAS3681, an androgen receptor antagonist, prevents drug resistance driven by aberrant androgen receptor signaling in prostate cancer

**DOI:** 10.1002/1878-0261.13641

**Published:** 2024-04-10

**Authors:** Shohei Yoshida, Daisuke Kajiwara, Masanao Seki, Manabu Tayama, Yuki Tanaka, Hiroya Mizutani, Ryoto Fujita, Keisuke Yamamura, Shigeo Okajima, Masanori Asai, Kazuhisa Minamiguchi

**Affiliations:** ^1^ Discovery and Preclinical Research Division Taiho Pharmaceutical Co., Ltd. Tsukuba Japan

**Keywords:** androgen receptor, antagonist, prostate cancer, TAS3681, tumor resistance

## Abstract

Second‐generation androgen receptor (AR) signaling inhibitors (ARSIs), such as abiraterone and enzalutamide, prolong the life of patients with castration‐resistant prostate cancer (CRPC). However, patients receiving ARSIs ultimately develop resistance through various complex mechanisms, including AR mutations, constitutively active AR‐splice variants (AR‐Vs), and AR overexpression. Here, we characterized a novel AR pure antagonist, TAS3681, which inhibits AR transcriptional activity and downregulates AR‐full length (AR‐FL) and AR‐Vs. TAS3681 reduced the protein levels of AR‐FL and AR‐Vs including AR‐V7 in enzalutamide‐resistant cells (SAS MDV No. 3‐14), *in vitro* and *in vivo*, showing strong antitumor efficacy in an AR‐V7‐positive xenograft model. In AR‐overexpressing VCaP (prostate cancer) cells, conversely to enzalutamide, TAS3681 effectively suppressed cell proliferation and downregulated AR expression. Importantly, TAS3681 blocked the transcriptional activity of various mutant ARs, including mutations F877L/T878A and H875Y/T878A, which confer resistance to enzalutamide, and V716M and H875Y mutations, which confer resistance to darolutamide. Our results demonstrate that TAS3681 suppresses the reactivation of AR signaling, which causes resistance to ARSIs, via a newly identified mechanism of action. Therefore, TAS3681 could be a new therapeutic option for CRPC treatment.

## Graphical abstract

The androgen receptor (AR) antagonist TAS3681 is effective against AR mutations, which leads to agonistic activity of AR signal inhibitors, and had an antitumor effect in an AR‐splice variant 7 (AR‐V7)‐positive enzalutamide‐resistant xenograft model. TAS3681 suppresses aberrant AR activation, including AR overexpression and expression of constitutively active nuclear‐localized AR‐V7, via downregulation of AR and AR‐V7.

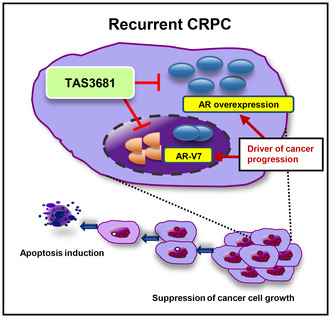

**Abbreviations**
Act D: actinomycin D
ADP: adenosine diphosphate
ADT: androgen deprivation therapy
AR: androgen receptor
AR‐FL: AR‐full length
ARSIs: androgen receptor signaling inhibitors
AR‐V7: AR‐splice variants 7
AR‐Vs: AR‐splice variants
ChIP: chromatin immunoprecipitation
CHX: cycloheximide
cPARP: cleaved‐poly (ADP‐ribose) polymerase
CRPC: castration‐resistant prostate cancer
DHT: dihydrotestosterone
DMSO: dimethyl sulfoxide
GAPDH: glyceraldehyde‐3‐phosphate dehydrogenase
KLK3: kallikrein‐related peptidase 3
LBD: ligand‐binding domain
mCRPC: metastatic castration‐resistant prostate cancer
PCa: prostate cancer
PSA: prostate‐specific antigen
qPCR: quantitative polymerase chain reaction
RTV: relative tumor volume
TMPRSS2: transmembrane protease serine 2
TV: tumor volume
UBE2C: ubiquitin‐conjugating enzyme E2 C
WB: western blotting

## 1 Introduction

Prostate cancer (PCa) is the second most common malignancy affecting men worldwide, with an estimated 1.41 million new cases a year, a frequency approaching that of lung cancer, which is the most common malignancy [1]. Initiation and progression PCa depend on androgen receptor (AR) activity. The standard treatment for advanced PCa is androgen deprivation therapy (ADT), which suppresses the transcriptional activity of ARs. ADT induces remission for 1–2 years in most patients, but cancer cells become resistant owing to the emergence of metastatic castration‐resistant prostate cancer (mCRPC) [2]. Earlier, first‐generation antiandrogens such as bicalutamide and flutamide were the standard treatment approaches against mCRPC; however, the partial agonist activity of the compounds has led to low drug efficacy and drug resistance in patients with mCRPC [3]. Over the past decade, enzalutamide, a second‐generation AR antagonist, and abiraterone, a CYP17A1 inhibitor, have proven effective against mCRPC and rapidly become standard therapies for advanced‐stage PCa [4, 5]. Despite their success, development of resistance against AR‐targeted therapies and maintenance of AR expression occurs in PCa [6, 7]. AR signaling can continue via several potential mechanisms despite treatment with antiandrogen therapies. These include mutations in the AR ligand‐binding domain (LBD), AR amplification, expression of AR‐splice variants (AR‐Vs), and alternative AR activation.

Here, we describe the characterization of TAS3681, an orally bioavailable AR antagonist that binds to the AR, inhibiting its nuclear translocation as well as its ligand‐dependent and ‐independent transcriptional activity. TAS3681 also inhibits clinically relevant AR mutations that confer resistance to first‐ and second‐generation AR signaling inhibitors (ARSIs).

## 2 Materials and methods

### 2.1 Cell culture

VCaP (RRID:CVCL_2235), COS‐7 (RRID:CVCL_0224), and HCC1806 (RRID:CVCL_1258) cells were purchased from the American Type Culture Collection (Rockville, MD, USA); HEK293 (RRID:CVCL_0045) cells were purchased from Health Science Research Resources Bank (Osaka, Japan); MIAPaCa‐2 (RRID:CVCL_0428), SK‐OV‐3 (RRID:CVCL_0532), MCF‐7 (RRID:CVCL_0031), and T‐47D (RRID:CVCL_0553) cells were purchased from Dainippon Sumitomo Pharma Co., Ltd. (Osaka, Japan). 22Rv1 (RRID:CVCL_1045) cells were obtained from European Collection of Cell Cultures (Salisbury, UK). LNCaP (RRID:CVCL_0395) cells were obtained from the Institute of Microbial Chemistry and the Microbial Chemistry Research Foundation (Tokyo, Japan). Enzalutamide‐resistant cells, SAS MDV No. 3‐14, were established as previously reported [8]; G418‐selected U‐2 OS tomato‐AR cells stably expressing red fluorescent protein (tdTomato) fused AR, and hygromycin B‐selected LNCaP cells stably expressing F877L mutant AR protein were established in‐house. All human cell lines were reauthenticated by means of short tandem repeat‐based DNA profiling and all experiments were performed with mycoplasma‐free cells. All cell lines were authenticated by STR profiling, performed at Biologica, Co. (Nagoya, Japan) after the study was completed. VCaP and COS‐7 cells were cultured in RPMI1640 containing 10% FBS. HEK293 were cultured in MEM containing 10% FBS. MCF‐7 cells were cultured in MEM containing 10% FBS, 0.1 mm NEAA, and 1 mm Na‐pyruvate. T‐47D cells were cultured in RPMI1640 containing 10% FBS and 0.2 I.U. insulin. MIAPaCa‐2 were cultured in DMEM including 10% cFBS and 2.5% horse serum. HCC1806 cells were cultured in RPMI1640, including 10 mm *N*‐2‐hydroxyethylpiperazine‐*n*′‐(2‐ethanesulfonic acid) (HEPES), 1 mm sodium pyruvate, and 10% FBS; SK‐OV‐3 cells were cultured in McCoy's 5A, including 15% FBS. The 22Rv1 cells were cultured in phenol red‐free RPMI 1640 (RPMI‐PR‐) containing 10% FBS. LNCaP cells and LNCaP cells stably expressing F877L mutant AR were cultured in RPMI 1640 medium containing 5% FBS. U‐2 OS tomato‐AR cells were cultured in McCoy's 5A medium containing 10% FBS. SAS MDV No. 3‐14 cells were maintained in RPMI‐PR‐ containing 5% FBS and 10 μm enzalutamide.

### 2.2 Plasmid and reagents

The vector plasmid expressing androgen receptor (AR; wild‐type, L702H, V716M, W742C, W742L, H875Q, H875Y, F877L, T878A, D891Y, Q903H, H875Y/T878A, F877L/T878A, T878A/S889G, and T878A/D891H) were obtained from Genewiz, Inc. (Beijing, China). The pGL4.36 [luc2P/MMTV/Hygro] and pGL4.75 [hRluc/CMV] vector plasmids were obtained from Promega Corporation (Madison, WI, USA). The pGLPE plasmid vector was generated by cloning the prostate‐specific antigen (PSA) promoter and enhancer fragments into the pGL3 plasmid vector [9]. TAS3681, 2‐chloro‐4‐[4‐[[5‐[2‐hydroxypropan‐2‐yl]pyridin‐2‐yl]amino]‐5,8‐dihydropyrido[3,4‐d]pyrimidin‐7(6H)‐yl]benzonitrile, and enzalutamide were synthesized by Taiho Pharmaceutical Co., Ltd. (Tsukuba, Japan) and obtained from AstaTech, Inc. (Bristol, PA, USA). Bicalutamide was obtained from Fidia Farmaceutici S.p.A. (Abano Terme, Italy). Apalutamide was obtained from Haoyuan Chemexpress Co., Ltd. (Shanghai, China). Darolutamide was obtained from MedChemExpress Co., Ltd. (Monmouth Junction, NJ, USA), TOK‐001 was obtained from Sundia MediTech Co., Ltd. (Shanghai, China). Dihydrotestosterone (DHT), cycloheximide (CHX), actinomycin D (Act D), and 17‐AAG were obtained from Sigma‐Aldrich (Tokyo, Japan). Camptothecin and paclitaxel were obtained from FUJIFILM Wako Pure Chemical Corporation (Osaka, Japan). Fetal bovine serum (FBS) and cFBS were obtained from Gibco (Grand Island, NY, USA) or HyClone (Logan, UT, USA); [^3^H]methyltrienolone was obtained from Perkin‐Elmer, Inc (Shelton, CT, USA). CellTiter‐Glo®, Bright‐Glo®, and Dual‐Glo® reagents were obtained from Promega KK (Tokyo, Japan). WST‐8 and 4′, 6‐diamidino‐2‐phenylindole (DAPI) solution were purchased from Dojindo Laboratories (Tokyo, Japan).

### 2.3 AR‐binding assay

Androgen receptor ligand‐binding studies were conducted *in vitro* with whole‐cell lysate, including wild‐type AR or T878A mutant AR [10], which were prepared according to previously reported methods [11, 12] and [^3^H]‐methyltrienolone. Cell lysates and [^3^H]‐methyltrienolone were combined with an incubation buffer (25 mm HEPES, pH 7.4, 10% glycerol, 1 mm EDTA, and 10 mm sodium molybdate) and incubated for 20 h at 4 °C. The bound radio‐ligand was then separated from the free radio‐ligand by vacuum filtration through 0.3% polyethyleneimine‐pretreated GF/B filter mats (Perkin‐Elmer, Inc.). The filter mats were washed four times with washing buffer (25 mm HEPES, pH 7.4, and 1 mm EDTA). The radioactivity was measured using a liquid scintillation counter. Non‐specific binding was estimated in the presence of 10 μm testosterone.

### 2.4 Luciferase reporter assay

Luciferase reporter assays were performed as described previously [9]. For the luciferase reporter assay of COS‐7 cells, the PSA‐promoter firefly luciferase plasmid, the *Renilla* luciferase reporter plasmid, and the wild‐type AR‐expressing plasmid were transfected using FuGENE® HD Transfection Reagent (Promega). For the luciferase reporter assay of LNCaP cells, the AR‐responsive firefly luciferase alone was transfected using the Amaxa™ Cell Line Nucleofector™ Kit (Lonza Ltd., Basel, Switzerland), as previously reported [9]. For the luciferase reporter assay of VCaP cells, the wild‐type AR‐expressing plasmid and AR‐responsive firefly luciferase reporter plasmid were transfected using Lipofectamine® 2000 or Lipofectamine® 3000 (Life Technologies Corporation, Waltham, MA, USA). For the luciferase reporter assay of HEK293 cells, AR‐responsive firefly luciferase reporter plasmid and the wild‐type AR or mutant AR‐expressing plasmid were transfected using FuGENE® HD Transfection Reagent. Luciferase activity was measured using Dual‐Glo® reagent or Bright‐Glo® reagent, following the manufacturer's instructions (Promega). AR transcriptional activity was measured in mammalian cells expressing AR‐dependent reporter constructs. DHT was used for the AR antagonist assays.

### 2.5 Proliferation assay

Proliferation assays were performed as described previously [9, 10]. For the proliferation assay of SAS MDV No. 3‐14 cells, the suspended cells in RPMI‐PR‐ containing 10% cFBS were seeded in 96‐well plates (3 × 10^3^ cells/well) and the plate incubated overnight in a humidified incubator with 5% CO_2_ at 37 °C for attachment. The cells were incubated with 3, 5, 7, and 10 μm of TAS3681 and enzalutamide for 3, 6, or 9 days in the absence of DHT. The culture medium was changed every 3 days. Cellular proliferation was assessed 3, 6, and 9 days after treatment by incubation with CellTiter‐Glo® reagent. Luminescence was measured using Multilabel Counter (Wallac Arvo‐HTS; Perkin‐Elmer). For proliferation assay of LNCaP and VCaP cells, each cell line was suspended in RPMI‐PR‐ containing 5% or 10% cFBS, respectively, and separately plated in 96‐well plates (3 × 10^3^ cells/well for LNCaP, 1 × 10^4^ cells/well for VCaP) and incubated overnight in a humidified incubator with 5% CO_2_ at 37 °C for attachment. They were treated with 0.003, 0.01, 0.03, 0.1, 0.3, 1, 3, and 10 μm of TAS3681, enzalutamide, bicalutamide, or apalutamide for 3 days in the presence of 0.1 μm DHT. A colorimetric growth assay using the WST‐8 solution was performed according to the manufacturer's instructions. For the proliferation assay of AR‐negative cells (MIAPaCa‐2, HCC1806, SK‐OV‐3 and DU145), each cell line was suspended in culture medium and seeded at a density of 3 × 10^3^ cells/well in 96‐well plates and incubated overnight in a humidified incubator with 5% CO_2_ at 37 °C for attachment. Cells were incubated with 0.003, 0.01, 0.03, 0.1, 0.3, 1, 3, 10 μm of TAS3681, or 0.1 μm of paclitaxel for 3 days. A crystal violet assay was performed as previously described [13].

### 2.6 Western blotting

Cell proteins were extracted using ice‐cold M‐PER Mammalian Protein Extraction Reagent (Thermo Fisher Scientific Inc., Waltham, MA, USA). Nucleic and cytoplasmic proteins were extracted according to the instructions of the corresponding extraction kit (Promega). Tumor proteins were extracted using ice‐cold T‐PER Mammalian Protein Extraction Reagent containing a protease inhibitor cocktail (Sigma‐Aldrich, Milan, Italy). Western blotting (WB) was performed according to the previously reported standard method to analyze protein expression [8]. First, equal amount of protein was loaded onto the gel. After running the gel, transferring the protein to a membrane (Bio‐Rad Laboratories, Inc. (Hercules, CA, USA) or Life Technologies Corporation), and incubating it with antibodies, the protein image was acquired using a chemiluminescence detection system (ImageQuant LAS‐3000 or ImageQuant LAS‐4010; GE Healthcare, Chalfont, UK or Amersham™ Imager 600 QC; Cytiva, Chalfont, UK). The primary antibodies used were as follows: human AR (clone D6F11; Cell Signaling Technology, Danvers, MA, USA or clone N‐20; Santa Cruz Biotechnology, Dallas, TX, USA), AR‐V7 (clone RM7; RevMAb Biosciences (Burlingame, CA, USA) and clone E3O8L; Cell Signaling Technology), estrogen receptor α (clone D8H8; Cell Signaling Technology), glucocorticoid receptor (clone D8H2; Cell Signaling Technology), progesterone receptor A/B (clone D8Q2J; Cell Signaling Technology), human glyceraldehyde‐3‐phosphate dehydrogenase (GAPDH; Catalog #2275‐PC‐100; Trevigen, Gaithersburg, MD, USA), HDAC1 (Catalog #2062), β‐tubulin (Catalog #2126), and cleaved‐poly (ADP‐ribose) polymerase (cPARP; Catalog #5625; Cell Signaling Technology).

### 2.7 Real‐time quantitative polymerase chain reaction (qPCR)

RNA was extracted using an RNeasy® mini kit (QIAGEN, Maryland, MD, USA) according to the manufacturer's instructions. Two and a half micrograms of total RNA were used for cDNA synthesis using the SuperScript VILO cDNA Synthesis Kit (Life Technologies Corporation). To detect indicated genes, each cDNA sample was amplified using TaqMan® Fast Universal PCR Master Mix (Life Technologies Corporation) using an ABI7900HT Real‐Time qPCR system (Thermo Fisher Scientific Inc.). The gene‐specific primers for cDNA and SYBR Green dye detection or TaqMan MGB or VIC probe were used (Table S1). Relative mRNA levels were calculated using the comparative threshold cycle method ($2^{–\Delta \Delta C_{t}}$), where the mRNA level of each gene of interest was normalized to that of the endogenous housekeeping gene *GAPDH*.

### 2.8 AR nuclear translocation

U‐2 OS tomato‐AR cells, suspended in RPMI‐PR‐ including 5% cFBS, were seeded on chamber slides (Thermo Fisher Scientific Inc.) or a 96‐well plate (1 × 10^3^ cells/well). The next day, the cells were treated with 10 μm of TAS3681, enzalutamide, and bicalutamide in the absence of DHT or were treated with 0.003, 0.01, 0.03, 0.1, 0.3, and 1 μm of TAS3681, enzalutamide, and bicalutamide in the presence of DHT for 2 h at 37 °C in a CO_2_ incubator. After fixation with a 4% PFA phosphate buffer solution, the cells were washed with PBS, and nuclear staining was performed using ProLong® Gold antifade mountant with DAPI. For imaging, coverslips were mounted onto cells, and a nuclear image of an AR was obtained using a confocal microscope (Olympus Corporation, Tokyo, Japan). For quantitative analysis, four images were acquired of each well, and measurement of AR fluorescence intensity in the nuclear region (N) and in the cytoplasmic region (C) was performed with the IN Cell Analyzer1000 (GE Healthcare) and the IN Cell Developer Toolbox software (GE Healthcare). The N/C Ratio Mean was calculated from data on the ratio between the nuclear intensity and cytoplasmic fluorescence intensity. The N/C Ratio Mean for each well was determined from the four images of the well.

### 2.9 Chromatin immunoprecipitation

Chromatin immunoprecipitation (ChIP) assay was performed as previously described [14]. VCaP cells grown in androgen‐depleted media with 10% cFBS were treated with 3 nm DHT or 10 μm enzalutamide and TAS3681 combined with 3 nm DHT for 4 h. The cells were cross‐linked for 10 min and processed for ChIP using an AR antibody (N‐20; Santa Cruz Biotechnology). Real‐time PCR quantification of immunoprecipitated kallikrein‐related peptidase 3 (*KLK3*) and transmembrane protease, serine 2 (*TMPRSS2*) enhancer is performed using SYBR Green dye detection and is shown as (% input). Primers (Sigma‐Aldrich Co. LLC, Irvine, CA, USA) used for qPCR of kallikrein‐related peptidase 3 (*KLK3*) [15] and transmembrane protease, serine 2 (*TMPRSS2*) [16] enhancers are shown in Table S1.

### 2.10 CHX and act D chase assay

CHX and Act D chase assays were performed as previously reported [17]. For the CHX chase assay, LNCaP and SAS MDV No. 3‐14 cells were cultured in RPMI‐PR‐ containing 5% cFBS. On the next day, LNCaP and SAS MDV No. 3‐14 cells were treated with 5 or 10 μm of TAS3681 or 1 μm of 17‐AAG for 0, 4, 8, or 24 h in the presence of 10 μg·mL^−1^ of CHX. Expression of AR protein was evaluated using WB. For the Act D chase assay, LNCaP and SAS MDV No. 3‐14 cells were cultured in RPMI‐PR‐ containing 5% cFBS. On the next day, LNCaP cells were treated with 5 or 10 μm of TAS3681 or 30 μg·mL^−1^ of CHX for 0, 4, 8, or 24 h in the presence of 5 μg·mL^−1^ Act D. Expression of AR protein and mRNA was evaluated using WB and gene expression analysis, respectively.

### 2.11 *In vivo* experiment

Five‐week‐old male CB‐17/Icr‐scid/scidJcl mice (SCID mice) were purchased from CLEA Japan, Inc. (Tokyo, Japan). The mice were castrated at 6 weeks of age and subcutaneously injected with SAS MDV No. 3‐14 cells [8]. To evaluate the antitumor activity, animals bearing SAS MDV No. 3‐14 cell xenografts were randomized on day 1 according to tumor volume, once the mean tumor volume had reached ~ 130–250 mm^3^, and administered with 7.5, 15, and 22.5 mg·kg^−1^ body weight of TAS3681 or vehicle control (0.5% hydroxypropyl methylcellulose; HPMC) twice daily by oral gavage for 14 days. The tumor volume and the body weight of mice were measured twice a week. The tumor volume (mm^3^) was defined as (*A* × *B* ^2^)/2, where *A* and *B* were the longest and the shortest tumor diameter, respectively. Relative tumor volume (RTV) on day 15 was calculated as the ratio of tumor volume (TV) on day 15 to that on day 0. On days 1 and 15, blood was collected from the facial vein, and serum was separated. Serum prostate‐specific antigen (PSA) concentrations were assessed using a PSA ELISA kit (Catalog #DKK300; R&D Systems, Minneapolis, MN, USA) according to the manufacturer's instructions, and the data were used to calculate the PSA ratio. The PSA ratio was calculated by dividing the PSA concentration of each sample on day 15 by that on day 1.

The mice were kept in the specific pathogen free animal facility with 12/12 h light/dark cycle. The animal facility is regularly tested for standard pathogens. Mice were fed no more than five per cage with free access to sterile water and food. Animal studies were reviewed and approved by the Institutional Animal Care and Use Committees at Taiho Pharmaceutical Co., Ltd. (Approval No. AE‐2016‐101) and carried out according to their guidelines for animal experiments.

### 2.12 Statistical analyses

Results are reported as mean ± standard deviation (SD) for *in vitro* experiments or mean ± standard error of the mean (SEM) for *in vivo* experiments. Statistical analyses and determination of the half‐maximal inhibitory concentration (IC_50_) values were performed using sas version 9.2 and exsus version 8.0.0 (CAC Exicare Corporation, Tokyo, Japan). Statistical significance was assessed using the Student's *t*‐test or Wilcoxon test for two groups and the Dunnett's test, nonparametric Dunnett's test, or Williams' test for more than three groups. In all analyses, statistical significance was set at *P* < 0.05.

## 3 Results

### 3.1 TAS3681 is a novel AR pure antagonist with AR downregulation activity

**Fig. 1:**
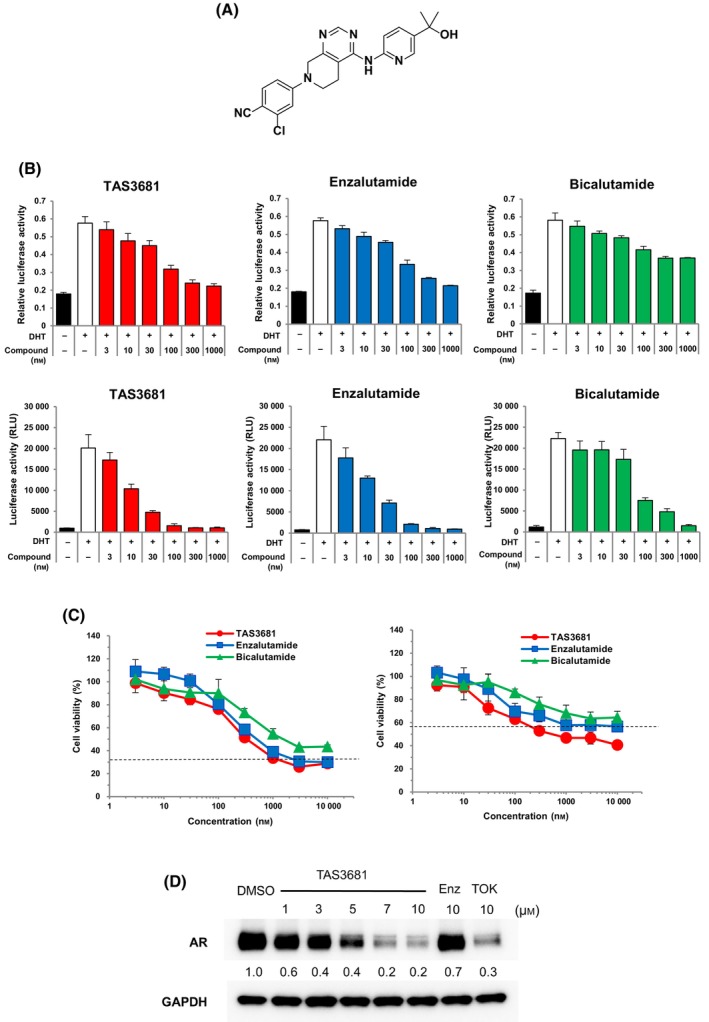
TAS3681 inhibits AR function and downregulates AR. (A) Chemical structure of TAS3681. (B) Effect of TAS3681, enzalutamide, and bicalutamide on the transcriptional activity of the wild‐type AR in COS‐7 cells (upper panel) or the T878A mutant AR in LNCaP cells (lower panel). COS‐7 cells were transfected with pGL4.36 luciferase (containing androgen‐dependent murine mammary tumor virus long terminal repeat), *Renilla* luciferase plasmids, and AR expression vector, and incubated for 1 day. LNCaP cells were transfected with the pGLPE luciferase plasmid and incubated for 1 day. COS‐7 cells and LNCaP cells were treated with various concentrations of TAS3681, enzalutamide, or bicalutamide with 1 nm DHT for COS‐7 or with 0.1 nm DHT for LNCaP, respectively, in steroid‐depleted medium for 1 day before luciferase activity measurements. Results are expressed as mean of triplicate wells ± SD. The IC_50_ values are presented in Table S3. (C) Inhibitory effect of TAS3681, enzalutamide, and bicalutamide on DHT‐induced proliferation of VCaP (left panel) and LNCaP cells (right panel). VCaP cells were treated with various concentrations of TAS3681, enzalutamide, and bicalutamide in the presence of DHT for 3 days. The dashed line indicates cell viability in the absence of DHT. Results are expressed as mean of sextuplicate wells ± SD. (D) AR and GAPDH protein expression in LNCaP cells treated with TAS3681, enzalutamide, and TOK‐001. LNCaP cells were treated with either the test compounds or DMSO for 1 day. Cell lysates were subjected to immunoblotting for AR and GAPDH to confirm equal loading. The numbers under each blot represent the relative expression value of the AR protein relative to that of DMSO‐treated cells. TOK‐001 was used as a positive control for the reduction in AR expression. A representative experiment (of 2) is shown. AR, androgen receptor; Enz, enzalutamide; TOK, TOK‐001.

Through modification of *N*‐arylpiperazine‐1‐carboxamide compounds [18], we discovered TAS3681 as a novel nonsteroidal compound in a screening campaign using AR transactivation assay, DHT‐dependent cell proliferation assay, and another cellular assay designed to detect compounds with the capacity to downregulate AR expression in LNCaP cells, and subsequent medicinal chemistry optimization (Fig. 1A). In the present study, a competitive AR‐binding assay was performed to investigate the binding affinity of TAS3681 to wild‐type AR and AR T878A mutants. The inhibition constant (*K*_i_) values of TAS3681 against wild‐type AR and AR T878A mutant were 7.39 and 23.8 nm, respectively (Table S2). These values were comparable to those of enzalutamide (7.11 nm for wild‐type AR and 22.6 nm for AR T878A mutant) and apalutamide (6.01 nm for wild‐type AR and 115.0 nm for AR T878A mutant) that were tested under the same conditions (Table S2). AR transactivation assays were performed using an AR‐responsive reporter along with a wild‐type AR in COS‐7 cells and VCaP cells that express wild‐type AR [19]. TAS3681 was a potent antagonist for wild‐type AR with IC_50_ values of 52.7 and 60.9 nm, respectively (Fig. 1B, Fig. S1 and Table S3). These values were comparable to those of enzalutamide (59.7 and 117.7 nm). Bicalutamide showed weaker antagonist activity (IC_50_ values of 429 and 662.5 nm for wild‐type AR) and could not completely block the transcriptional activity of wild‐type AR even at 1000 nm because of its partial agonist profile [20] (Fig. 1B, Fig. S1 and Table S3). Moreover, AR transactivation assays were performed using an AR‐responsive reporter along with LNCaP that express T878A mutant [21]. TAS3681 also shown potent antagonist activity (IC_50_ value of 10.1 nm) similar to enzalutamide (IC_50_ value of 12.5 nm) (Fig. 1B and Table S3). To determine whether the anti‐AR transcriptional activity of TAS3681 is accompanied by reduced cell proliferation, the number of viable VCaP and LNCaP cells was quantified after incubation with TAS3681. TAS3681 suppressed the DHT‐induced proliferation of VCaP cells in a dose‐dependent manner, with an IC_50_ of 170 nm, which was comparable to that of enzalutamide (IC_50_ of 180 nm) (Fig. 1C and Table S4). TAS3681, as well as enzalutamide and bicalutamide, also dose‐dependently suppressed LNCaP cell proliferation, with an IC_50_ of 18 nm. Enzalutamide and bicalutamide had IC_50_ values of 55 and 340 nm, respectively (Fig. 1C and Table S4). Consistent with the results of the antagonist assay, bicalutamide showed weaker inhibition of cell proliferation in VCaP and LNCaP, and the IC_50_ values for bicalutamide in each cell line were approximately 3‐ and 19‐fold higher, respectively, than those for TAS3681. Therefore, TAS3681 inhibited DHT‐induced cell proliferation more potently than bicalutamide, and with activity similar to enzalutamide.

No significant effect of TAS3681 was observed on the viability of AR‐negative cells, such as MIAPaCa‐2 (human pancreatic carcinoma), HCC1806 (human breast cancer), SK‐OV‐3 (human ovarian cancer), and DU145 (human prostate cancer) indicating that the antiproliferative effect of TAS3681 in AR‐positive PCa cells is mediated through AR antagonism (Figs S2 and S3).

We evaluated the effect of TAS3681 on AR protein levels using WB with an AR antibody‐N‐terminal. LNCaP cells were treated with TAS3681 for 1 day; TAS3681, but not enzalutamide, reduced AR expression in a dose‐dependent manner (Fig. 1D). Similar results were observed for VCaP cells (Fig. S4). In addition, TAS3681 downregulated AR protein, but had little effect on the protein levels of other nuclear receptors, such as estrogen receptor, glucocorticoid receptor, and progesterone receptor (Fig. S5). Overall, the findings suggest that TAS3681 has the same potential as enzalutamide, a representative second‐generation compound, as an AR pure antagonist, but also has unique AR reduction activity.

### 3.2 TAS3681 functions as a pure antagonist for various AR mutants

**Fig. 2:**
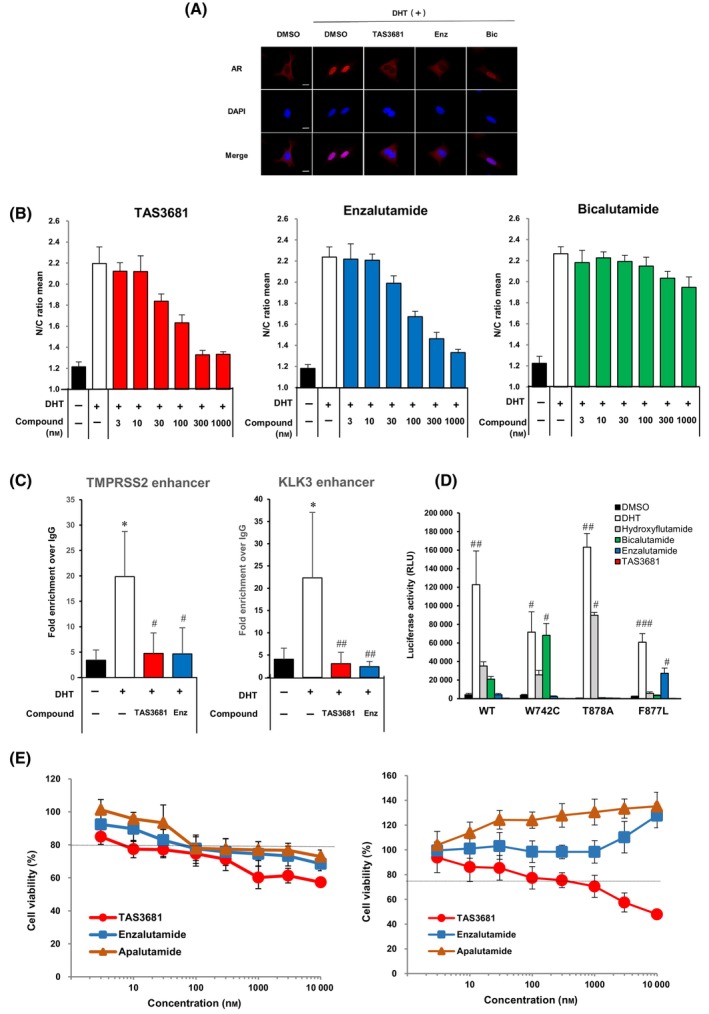
TAS3681 impairs nuclear translocation and DNA binding of AR in prostate cancer cells. (A) Representative confocal microscopic images of the U‐2 OS tomato‐AR cells stably expressing tdTomato fused AR [37] pretreated for 0.5 h with 1 μm of TAS3681, enzalutamide, or bicalutamide, followed by treatment for 4 h with the compounds in the presence of 0.1 nm DHT in the steroid‐depleted medium. The fluorescence images are cropped from photographs taken at a magnification of 10× using an objective lens and a dichroic mirror. The nuclei were stained with DAPI (blue). A representative experiment (of 2) is shown. Scale bars: 10 μm. (B) U‐2 OS tomato‐AR cells were pretreated for 0.5 h with 1 μm TAS3681, enzalutamide, or bicalutamide, followed by treatment for 4 h with the compounds in the presence of 0.1 nm DHT in a steroid‐depleted medium and imaged with an imaging cytometer. The ordinate represents the ratio of fluorescent intensities of nuclear (shown as N) to cytoplasmic (shown as C) as the N/C ratio. Results are expressed as the mean of sextuplicate wells ± SD. (C) The binding of AR on the enhancer of *KLK3* or *TMPRSS2* in VCaP cells treated for 4 h with 3 nm DHT alone versus 3 nm DHT plus 10 μm enzalutamide or 3 nm DHT plus 10 μm TAS3681 was analyzed by ChIP‐qPCR assay. Results are expressed as the mean of quadruplicate samples ± SD. *P < 0.05 versus DMSO‐treated cells using Wilcoxon test, ^#^ *P <* 0.05; ^##^ *P* < 0.01 versus DHT‐treated cells using Dunnett's test. (D) Agonist activities of 10 μm hydroxyflutamide, bicalutamide, enzalutamide, and 3 μm TAS3681 against wild‐type or mutant ARs; HEK293 cells were transfected with expression vector of wild‐type or mutant AR and pGL4.36 luciferase plasmid and incubated for 1 day. To analyze the T878A mutant AR, LNCaP cells were transfected with pGLPE luciferase plasmid and incubated for 1 day. Each cell was treated with the indicated compounds in steroid‐depleted medium for 1 day before luciferase activity measurements. Results are expressed as mean of sextuplicate wells ± SD. ^#^ *P <* 0.05; ^##^ *P <* 0.01; ^###^ *P <* 0.001 versus DMSO‐treated cells using Dunnett's test. (E) Inhibitory effects of TAS3681, enzalutamide, and apalutamide on DHT‐induced proliferation of LNCaP (left panel) and F877L‐expressing LNCaP cells (right panel). The cells were incubated for 6 days with various concentrations of TAS3681, enzalutamide, and apalutamide in the presence of 0.1 nm DHT. The dashed line indicates cell viability in the absence of DHT. Results are expressed as mean of sextuplicate wells ± SD. A representative experiment is shown. AR, androgen receptor; Bic, bicalutamide; Enz, enzalutamide; *KLK3*, kallikrein‐related peptidase 3; *TMPRSS2*, transmembrane protease serine 2.

Androgen receptor movement from the cytoplasm to the nucleus upon androgen binding is an essential step in AR‐mediated gene transcription. Second‐generation small‐molecule ARSIs, including enzalutamide, block the nucleo‐cytoplasmic translocation of AR and DNA binding without any AR agonist activity. As shown in Fig. 2A, AR was predominantly localized in cytoplasm in the absence of androgen, and DHT addition increased the nuclear/cytoplasmic (N/C) ratio of fluorescent intensity (N/C ratio: approximately 2.2), indicating the translocation of AR from the cytoplasm to the nucleus (Fig. 2B).

TAS3681 potently and dose‐dependently blocked AR nuclear translocation in U‐2 OS tomato‐AR cells stimulated with DHT (Fig. 2B). Enzalutamide also showed dose‐dependent inhibition of AR nuclear translocation (Fig. 2B). Conversely, bicalutamide showed little inhibition of AR nuclear translocation in those cells (Fig. 2A,B). The IC_50_ of TAS3681 for blocking DHT‐induced AR nuclear translocation was estimated to be 63.7 nm, whereas that of enzalutamide was 103 nm (Table S5). Bicalutamide did not suppress AR nuclear translocation (Table S5). We also examined the effect of antiandrogens on AR subcellular localization in the absence of androgen. Nuclear translocation of AR was not observed after treating cells with TAS3681 or enzalutamide (Fig. S6A). The N/C ratios of cells treated with 10 μm TAS3681 or enzalutamide were calculated to be 1.360 and 1.325, respectively, which were similar to those of cells treated with DMSO indicating that TAS3681 and enzalutamide do not induce AR nuclear translocation and have a pure antagonist profile (Fig. S6B). In contrast, nuclear translocation of AR was observed after the treatment of cells with 10 μm bicalutamide, as previously reported [20]. The N/C ratio of cells treated with bicalutamide was 1.858, indicating that bicalutamide induces AR nuclear translocation and has partial agonist activity (Fig. S4B).

Next, we examined the effect of TAS3681 on AR binding to DNA in PCa cells. ChIP‐qPCR assays revealed that AR was efficiently recruited to the enhancer regions of *KLK3* and *TMPRSS2* in VCaP cells in the presence of DHT. Treatment with TAS3681 or enzalutamide impaired the AR binding to these regions (Fig. 2C). This finding suggests that TAS3681 blocks the nucleo‐cytoplasmic translocation of AR and DNA binding without any AR agonist activity.

Mutations in the AR are known to promote resistance to antiandrogen therapies, including enzalutamide [22, 23]. AR mutations are more common in patients progressing to these therapies than in responders [24]. The effects of antiandrogens on mutant ARs such as T878A, AR W742C, and AR F877L were studied using cell‐based transient transactivation assays with an androgen‐responsive luciferase reporter gene construct. In the absence of DHT, agonist mode transactivation assays showed that enzalutamide acted as an agonist for the AR F877L mutant, whereas bicalutamide and hydroxyflutamide were agonistic for mutants AR W742C and AR T878A, respectively (Fig. 2D). However, TAS3681 alone did not show agonistic profiles for all tested mutant ARs in the absence of DHT (Fig. 2D). We obtained similar results by transactivation assays in the presence of DHT, and only TAS3681 functioned as a pure antagonist for all tested mutant ARs (Fig. S7). Moreover, we expanded the list of functionally characterized AR‐LBD mutants with an additional 10 variants (mutated ARs L702H, V716M, W742L, H875Q, H875Y, D891Y, Q903H, H875Y/T878A, T878A/S889G, and T878A/D891H) [25, 26]. In addition to the three AR mutations tested earlier, we evaluated the response of these 10 AR mutants to increasing concentrations of DHT to determine the optimal concentration for each AR mutant, following a process similar to that for other mutants. The effects of antiandrogens (TAS3681, enzalutamide, apalutamide, and darolutamide) on the transactivation of 13 AR mutants were studied, and IC_50_ values were calculated (Fig. S8 and Table S6). Enzalutamide and apalutamide showed agonistic effects on the mutated ARs F877L/T878A, H875Y/T878A, and F877L (Fig. S8 and Table S6). While darolutamide showed an agonistic effect on the mutated ARs V716M and H875Y, TAS3681 did not show an agonistic effect on any additional mutant ARs in this study (Fig. S8 and Table S6). Next, we investigated the effectiveness of TAS3681 in inhibiting the proliferation of LNCaP cells stably expressing F877L AR (F877L AR cells). TAS3681 treatment in the presence of DHT potently and dose‐dependently suppressed the proliferation of both the parent and F877L AR cells (Fig. 2E). Enzalutamide and apalutamide suppressed the proliferation of parent cells but failed to suppress the proliferation of F877L AR cells at any concentration, as previously reported (Fig. 2E) [7]. Taken together, these findings suggest that TAS3681 functions as an AR pure antagonist against wild‐type and clinically relevant LBD mutations.

### 3.3 TAS3681 reduces both AR‐FL and AR‐V7 protein levels and suppresses the proliferation of enzalutamide‐resistant PCa cells

**Fig. 3:**
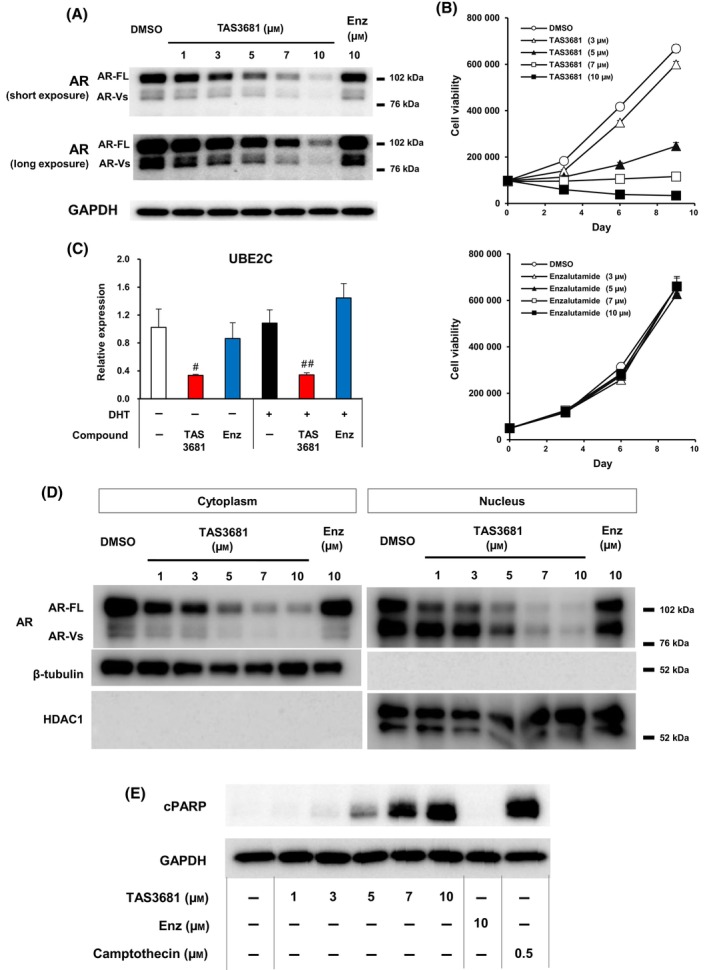
TAS3681 reduces both AR‐full length (AR‐FL) and AR‐splice variant 7 (AR‐V7) protein levels and suppresses the proliferation of enzalutamide‐resistant prostate cancer cells. (A) Downregulation of AR‐FL and AR‐Vs protein expression in SAS MDV No. 3‐14 cells exposed to TAS3681. SAS MDV No. 3‐14 cells were exposed to TAS3681 or enzalutamide for 1 day at the indicated concentrations. Cell lysates were subjected to immunoblotting for AR (N‐terminal) and GAPDH to confirm equal protein loading. Then, AR‐FL and AR‐V proteins were detected by assessment of luminescence over a short or long period, respectively. A representative experiment (of 3) is shown. (B) Effects of TAS3681 (upper panel) and enzalutamide (lower panel) on the growth of SAS MDV No. 3‐14 cells. SAS MDV No. 3‐14 cells were treated with TAS3681 or enzalutamide at the indicated concentrations for 3, 6, or 9 days in an androgen‐free medium. Results are expressed as mean of triplicate wells ± SD. (C) Effect of TAS3681 on AR‐V7‐related target genes in SAS MDV No. 3‐14 cells. SAS MDV No. 3‐14 cells were cultured under androgen‐deprived conditions for 1 day and treated with DMSO or 7.5 μm of TAS3681 or enzalutamide in the absence or presence of DHT for 1 day. Total RNA was extracted from the cells, and cDNA was synthesized. The mRNA expression of *UBE2C* in SAS MDV No. 3‐14 cells was analyzed by RT‐qPCR analysis. The level of each target gene was normalized to the corresponding expression of GAPDH, and the relative mRNA expression in the DMSO‐treated cells without DHT was set as 1. Results are expressed as mean of triplicate wells ± SD. Statistical significance was analyzed by Dunnett's test, ^#^ *P* < 0.05 versus DMSO‐treated cells in the absence of DHT; ^##^ *P* < 0.01 versus DMSO‐treated cells in the presence of DHT. (D) Effect of TAS3681 on AR‐FL and AR‐Vs protein levels in nuclear and cytoplasmic fractions. SAS MDV No. 3‐14 cells grown in androgen‐depleted medium were treated with TAS3681, enzalutamide, or vehicle control for 1 day. Levels of cytoplasmic and nuclear AR‐FL and AR‐Vs were assessed by WB analysis. WB of the nuclear and cytoplasmic fractions was performed with anti‐HDAC1 (nuclear) and anti‐β‐tubulin (cytoplasmic) antibodies to confirm successful cell fractionation. A representative experiment (of 3) is shown. (E) Evaluation of cPARP protein expression in SAS MDV No. 3‐14 cells by TAS3681, enzalutamide, or camptothecin. SAS MDV No. 3‐14 cells were treated with the indicated concentrations of TAS3681, enzalutamide, or camptothecin. Cell lysates were prepared following 1 day of treatment and were subjected to immunoblotting for cPARP, as well as for GAPDH, to confirm equal loading. A representative experiment (of 2) is shown. AR, androgen receptor; cPARP, cleaved‐poly (ADP‐ribose) polymerase; Enz, enzalutamide; TAS, TAS3681; UBE2C, ubiquitin‐conjugating enzyme E2 C.

Since TAS3681 showed AR reduction activity in a previous cell‐based study, we examined whether TAS3681 also reduces the expression of both AR‐FL and AR‐V7 in SAS MDV No. 3‐14 cells [8]. As shown in Fig. 3A, Figs S9 and S10, TAS3681 effectively and dose‐dependently reduced not only AR‐FL but also AR‐V7 in SAS MDV No. 3‐14 cells. TAS3681 also reduced the protein levels of both ARs in 22Rv1 cells, another AR‐V7 positive enzalutamide‐resistant cell line (Fig. S11) [27]. TAS3681 effectively and dose‐dependently suppressed the proliferation of SAS MDV No. 3‐14 cells expressing AR and AR‐V7 (Fig. 3B). In contrast, enzalutamide did not suppress the growth of SAS MDV No. 3‐14 cells at any of the concentrations tested (Fig. 3B).

Next, to examine whether TAS3681 affected the transcriptional activity of AR‐V7, real‐time qPCR analysis was conducted to quantify mRNA levels of AR‐V7‐dependent endogenous genes, including *CDC20*, *CDK1*, *CCNA2*, and *UBE2C*, in SAS MDV No. 3‐14 cells [28]. DHT did not affect the mRNA levels of the genes in SAS MDV No. 3‐14 cells (Fig. 3C and Fig. S8). TAS3681 considerably decreased the expression of AR‐V7 target genes in the presence and absence of DHT, whereas enzalutamide did not (Fig. 3C and Fig. S12). Next, to further explore the subcellular locations of AR‐FL and AR‐Vs, cytoplasmic and nuclear protein fractions of SAS MDV No. 3‐14 cells were analyzed. Under androgen‐depleted conditions, AR‐FL was detected in both the cytoplasm and nucleus, whereas AR‐Vs were localized predominantly in the nucleus, as previously reported [29]. TAS3681 reduced the expression of both AR‐Vs and AR‐FL within their respective localizations (Fig. 3D). In contrast, enzalutamide did not affect the expression of AR‐Vs in the nucleus and that of AR‐FL in the cytoplasm and nucleus (Fig. 3D).

The downregulation of AR‐V7 by antisense oligonucleotides is accompanied by suppression of androgen‐independent cell growth and apoptosis induction in PCa cells [30]. We found through WB analysis that cPARP, an apoptosis‐related protein, was expressed in SAS MDV No. 3‐14 cells treated with TAS3681 (Fig. 3E). In contrast, cPARP was not expressed after enzalutamide treatment (Fig. 3E). Altogether, TAS3681 suppressed the proliferation and induced apoptosis accompanied by downregulation of AR‐FL and AR‐V7 in enzalutamide‐resistant SAS MDV No. 3‐14 cells.

### 3.4 TAS3681 reduces AR protein levels and suppresses AR transactivation and growth of AR‐overexpressing enzalutamide‐resistant cells

**Fig. 4:**
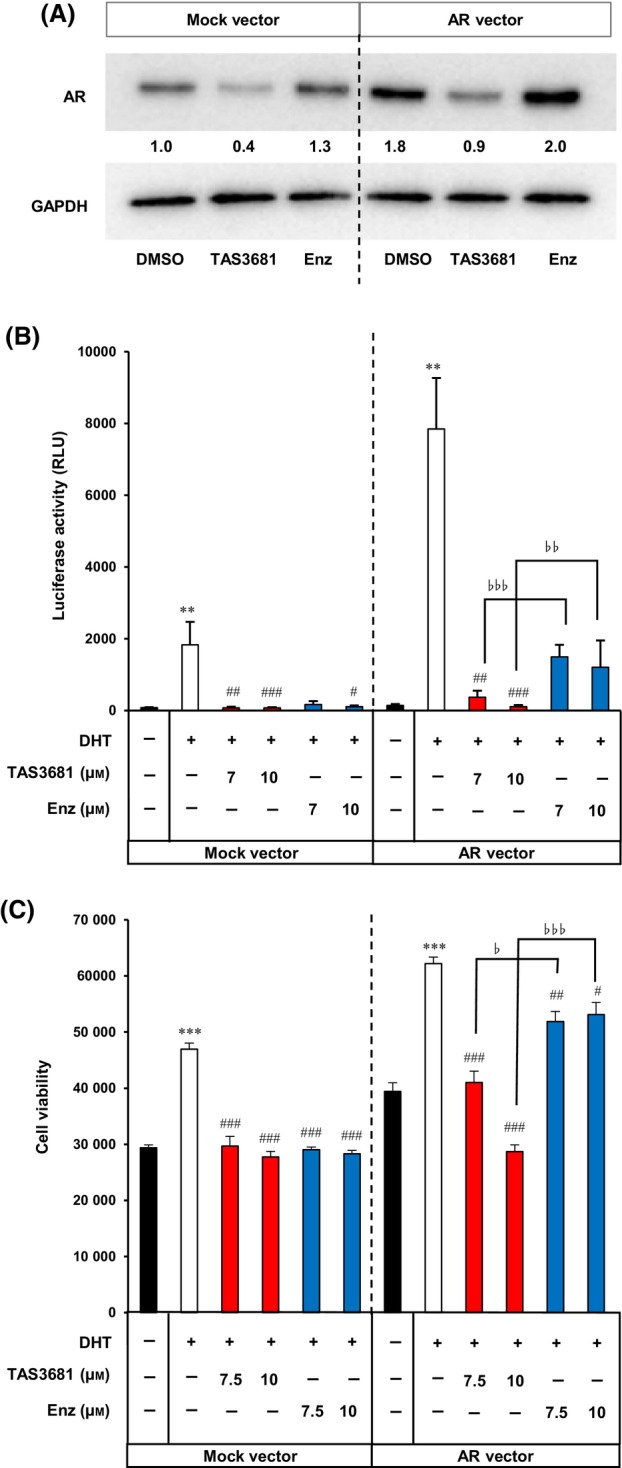
TAS3681 reduces AR protein level and suppresses AR transactivation and the growth of AR‐overexpressing prostate cancer cells. (A) Effect of TAS3681 on AR protein levels in AR‐overexpressing VCaP cells. VCaP cells were transfected with a mock vector or an AR expression vector and treated with the indicated compounds (10 μm) for 1 day. Cell lysates were subjected to immunoblotting for AR and GAPDH to confirm equal loading. Numbers below the AR band indicate AR protein expression relative to that in DMSO control cells transfected with a mock vector. A representative experiment (of 3) is shown. (B) Effects of TAS3681 and enzalutamide on AR transcriptional activity in AR‐overexpressing VCaP cells. VCaP cells were transfected with a mock vector or an AR expression vector, and the pGL4.36 luciferase plasmid and incubated for 1 day. The cells were treated with TAS3681 or enzalutamide at the indicated concentrations with 3 nm DHT in steroid‐depleted medium for 1 day before luciferase activity measurements. Results are expressed as mean ± SD. **P < 0.001 versus DMSO‐treated cells using Wilcoxon test, ^#^ *P* < 0.05; ^##^ *P* < 0.01; ^###^ *P* < 0.001 versus DHT‐treated cells using Dunnett's test, ^♭♭^ *P* < 0.01; ^♭♭♭^ *P* < 0.001 by Student's *t*‐test. (C) Effects of TAS3681 and enzalutamide on the growth of AR‐overexpressing VCaP cells. VCaP cells were transfected with a mock vector or AR expression vector, followed by incubation for 3 days with TAS3681 or enzalutamide at the indicated concentrations in the presence of 0.3 nm DHT. Results are expressed as mean of triplicate wells ± SD. ***P < 0.001 versus DMSO‐treated cells using Student's *t*‐test, ^#^ *P <* 0.05; ^##^ *P <* 0.01; ^###^ *P <* 0.001 versus DHT‐treated cells using Dunnett's test, ^♭^ *P* < 0.05; ^♭♭♭^ *P* < 0.001 by Student's *t*‐test. AR, androgen receptor; Enz, enzalutamide.

Androgen receptor overexpression and amplification have been reported as mechanisms of enzalutamide resistance [24, 26]. Next, we examined the effect of AR overexpression on the AR inhibitory activity of TAS3681 and enzalutamide. VCaP cells transfected with the wild‐type AR showed higher AR protein levels, and as a result, AR transcriptional activation and proliferation were more efficiently enhanced by DHT in AR‐overexpressing cells than in parental cells (Fig. 4A–C). Enzalutamide completely blocked DHT‐induced AR transcriptional activation and proliferation in parental cells but did not completely suppress them in AR‐overexpressing cells (Fig. 4A–C). In contrast, TAS3681 effectively blocked DHT‐induced AR transcriptional activation and proliferation in both parental and AR‐overexpressing cells. These observations of TAS3681 were consistent with the attenuation of AR levels in AR‐overexpressing cells treated with TAS3681 (Fig. 4A–C). The addition of AR‐lowering effects to AR antagonists might be an effective strategy to address the issues of AR amplification and overexpression.

### 3.5 TAS3681 downregulates both AR‐FL and AR‐V7 protein levels at the translational level

**Fig. 5:**
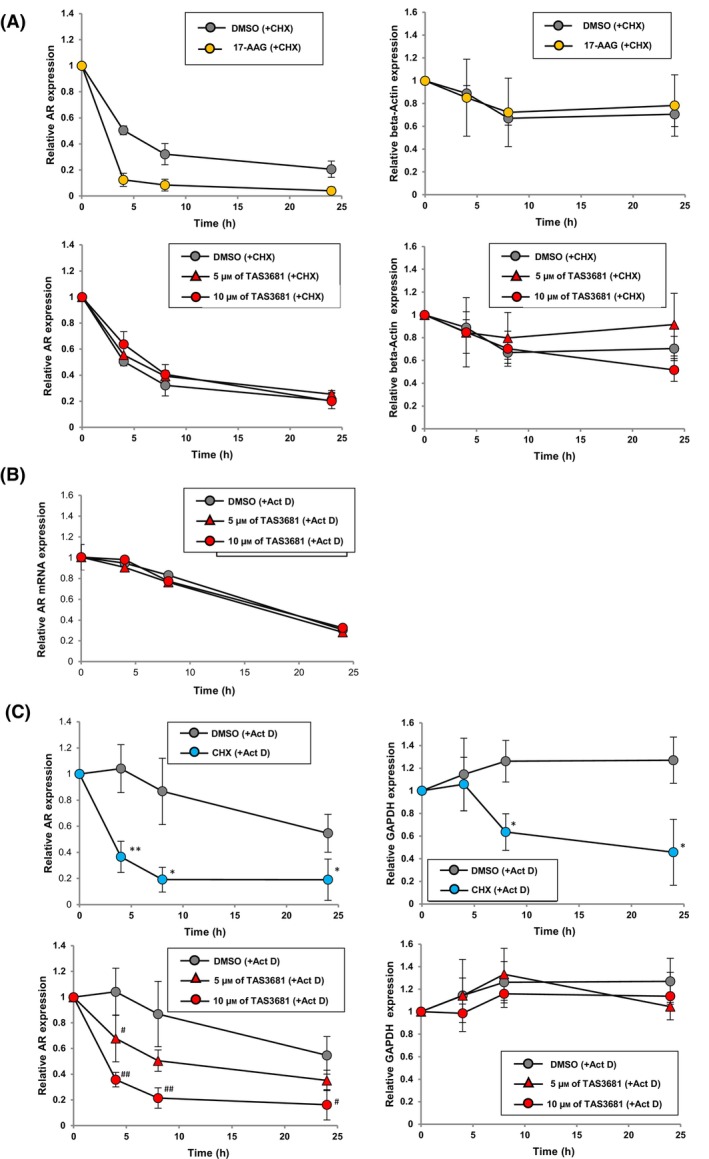
TAS3681 downregulates AR protein levels at the translational level. (A) Effect of TAS3681 and 17‐AAG on the stability of AR and β‐actin proteins in LNCaP cells. LNCaP cells were treated with TAS3681 at concentrations of 5 and 10 μm or with 17‐AAG at a concentration of 1 μm for 0, 4, 8, or 24 h in the presence of CHX (10 μg·mL^−1^). The expression levels of AR and β‐actin proteins were evaluated by WB analysis. The expression level of AR or β‐actin protein in the 0 h sampling group was set to 1 to evaluate the relative expression of AR or β‐actin protein at each sampling time. Results are expressed as mean of triplicate wells ± SD. (B) Effect of TAS3681 on the stability of *AR* mRNA in LNCaP cells. LNCaP cells were treated with TAS3681 at a concentration of 5 or 10 μm for 0, 4, 8, or 24 h in the presence of Act D (5 μg·mL^−1^). The expression of *AR* mRNA was evaluated by RT‐PCR. The expression level of *AR* mRNA in the control group (0 h sampling group) was set to 1 to evaluate the relative expression of *AR* mRNA at each sampling time. Results are expressed as mean of triplicate wells ± SD. (C) Effect of TAS3681 and CHX on AR and GAPDH protein expression in LNCaP cells in the presence of Act D. LNCaP cells were treated with TAS3681 at a concentration of 5 and 10 μm or with CHX at a concentration of 30 μg·mL^−1^ for 0, 4, 8, or 24 h in the presence of Act D (5 μg·mL^−1^). The expression of AR and GAPDH proteins was evaluated by WB analysis. Results are expressed as mean of triplicate wells ± SD. The expression levels of AR or GAPDH protein in the 0 h sampling group were set to 1 to evaluate the relative expression of AR or GAPDH protein at each sampling time. *P < 0.05, **P < 0.01 versus DMSO‐treated cells using Student's *t*‐test; ^#^ *P <* 0.05, ^##^ *P* < 0.01 versus DMSO‐treated cells using Dunnett's test. AR, androgen receptor; Act D, actinomycin D; CHX, cycloheximide.

Next, we investigated whether the mechanism of AR downregulation by TAS3681 caused an increase in the rate of AR protein degradation or a decrease in AR protein synthesis. A CHX chase assay was used to analyze AR protein stability. Treatment of LNCaP cells with TAS3681 in the presence of CHX did not increase the rate of AR degradation as well as β‐actin degradation compared with the vehicle control, in contrast to HSP90 inhibitor 17‐AAG (Fig. 5A and Fig. S13). Next, we examined *AR* mRNA and protein levels in the presence of Act D, an inhibitor of mRNA synthesis. *AR* mRNA levels were evaluated by RT‐qPCR after treatment with TAS3681 in the presence of Act D. Treatment with TAS3681 did not significantly reduce *AR* mRNA level compared to vehicle treatment; therefore, TAS3681 did not affect *AR* mRNA stability (Fig. 5B). The AR protein level was evaluated using WB analysis after the treatment of TAS3681 in the presence of Act D. The results showed that the AR protein level reduced over time after TAS3681 treatment (Fig. 5C lower left panel and Fig. S14). GAPDH protein expression did not decrease over time after TAS3681 treatment (Fig. 5C lower right panel and Fig. S14). Conversely, a non‐selective protein synthesis inhibitor, CHX, promoted the reduction of both AR and GAPDH levels in the presence of Act D (Fig. 5C upper panel and Fig. S14). TAS3681 did not show significant effect on *AR* mRNA levels in LNCaP cells compared to the vehicle (Fig. S15). Similarly, we investigated the mechanism of AR‐Vs and AR‐V7 downregulation by TAS3681 using SAS MDV No. 3‐14 cells. Treatment with TAS3681 did not prompt degradation of AR‐Vs protein in the presence of CHX. In the presence of Act D, AR‐V7 mRNA stability was not affected by TAS3681, while AR‐Vs protein level was reduced by TAS3681 compared to vehicle treatment (Fig. S16A–C). TAS3681 did not show significant effect on AR‐V7 mRNA levels in SAS MDV No. 3‐14 cells compared to the vehicle (Fig. S17). Considering the present findings, it can be deduced that TAS3681 reduces the rates of AR and AR‐Vs protein synthesis.

### 3.6 TAS3681 suppresses tumor growth in enzalutamide‐resistant PCa xenograft

**Fig. 6:**
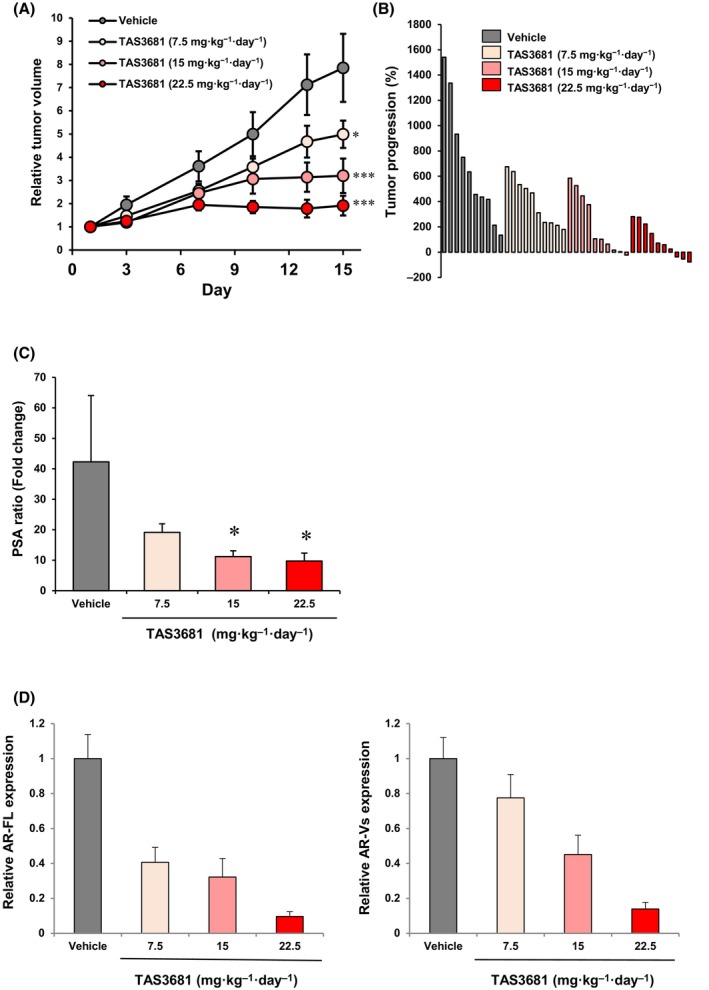
TAS3681 suppressed tumor growth in enzalutamide‐resistant prostate cancer xenograft *in vivo*. (A) Antitumor effect of TAS3681 in castrated SCID mice implanted with human prostate cancer SAS MDV No. 3‐14 cells. SAS MDV No. 3‐14 cells were implanted subcutaneously into the right flank of castrated SCID mice. Vehicle or TAS3681 (7.5, 15, or 22.5 mg·kg^−1^·day^−1^) was administered orally twice a day for 14 days. Results are expressed as mean ± SEM (*n* = 10). *P < 0.025, ***P < 0.0005 versus vehicle group using Williams' test. (B) Individual tumor progression with the administration of vehicle or TAS3681 in castrated SCID mice bearing human prostate cancer SAS MDV No. 3‐14 cells. Waterfall plots show tumor progression in each of the mice between days 1 and 15. A representative experiment (of 2) is shown. (C) Effect of TAS3681 on serum PSA levels in castrated SCID mice bearing SAS MDV No. 3‐14 human prostate cancer xenografts. On days 1 or 15, blood was collected, and serum was prepared. The PSA ratio indicated a fold change from the baseline after 14 days of treatment. Results are expressed as mean ± SEM (*n* = 10 mice in each group except for vehicle group; *n* = 9). A representative experiment (of 2) is shown. *P < 0.025 versus vehicle group using Williams' test. (D) Downregulation of AR‐FL (left) and AR‐Vs protein (right) expression in tumors of castrated SCID mice after treatment with TAS3681. Tumors were harvested on day 15, and tumor tissue lysates were subjected to immunoblotting for AR‐FL, AR‐Vs, and β‐actin. The expression of AR‐FL protein or AR‐Vs protein in tumor tissue was normalized to that of β‐actin, and the relative AR‐FL and AR‐Vs expression in the vehicle‐treated group was set as 1. Results are expressed as mean ± SEM (*n* = 10). AR, androgen receptor; AR‐FL, androgen receptor full length; AR‐Vs, androgen receptor splice variants; PSA, prostate‐specific antigen.

To assess the *in vivo* efficacy of TAS3681 in an enzalutamide‐resistant CRPC mouse model, castrated male SCID mice with subcutaneously injected SAS MDV No. 3‐14 cells were treated orally with TAS3681 twice daily for 14 days [8]. The half‐life of TAS3681 in mice was not optimal (3.63 h); therefore, TAS3681 was orally dosed using bids. The ratios of the mean RTV in each TAS3681‐treated group (7.5, 15, or 22.5 mg·kg^−1^·day^−1^) to those in the vehicle control group (T/C ratio, %) were 64%, 41%, and 24%, respectively. At all doses, the difference in the mean RTV between the vehicle group and each TAS3681 group was statistically significant (*P* < 0.025) (Fig. 6A and Fig. S18). Tumor regression was observed in one of ten mice treated with 15 mg·kg^−1^·day^−1^ of TAS3681, and three of ten mice treated with 22.5 mg·kg^−1^·day^−1^ of TAS3681 (Fig. 6B). Administration of TAS3681 caused a dose‐dependent decrease in serum PSA levels in castrated SCID mice bearing SAS MDV No. 3‐14 tumors. At 15 and 22.5 mg·kg^−1^·day^−1^ of TAS3681, the difference in mean fold PSA change from baseline after 14 days of treatment between the vehicle group and both TAS3681 groups was statistically significant (*P* < 0.025) (Fig. 6C and Fig. S19). In the present study, the expression of AR‐FL and AR‐Vs proteins in harvested tumors (on day 15) was analyzed by WB. As shown in Fig. 6D and Fig. S20, the expression of AR‐FL and AR‐Vs in xenograft tumors was dose‐dependently downregulated by TAS3681. Altogether, these results indicate that TAS3681 exerts significant antitumor effects against enzalutamide‐resistant PCa xenografts, accompanied by a reduction of AR‐FL and AR‐Vs levels in tumors.

## 4 Discussion

The management of CRPC has changed considerably over the past few decades [31]. Remarkable progress has been made in targeting persistent AR activation with second‐generation hormonal therapies (enzalutamide and abiraterone acetate) in recent years, but resistance to these agents limits therapeutic efficacy in many patients [32]. Thus, there is an urgent need to identify novel therapeutics. Several major resistance pathways are involved in androgen signaling, including AR overexpression and amplification, AR mutant expression, intratumoral and adrenal androgen production, and constitutively active AR‐V7 [33]. AR‐V7 has been reported to lack LBDs, be constitutively activated in an androgen‐independent manner, and correlate with poor prognosis. AR‐V7 is a constitutively active transcription factor that lacks the LBD but retains the N‐terminal and DNA‐binding domain; therefore, it can circumvent the limitations of current ARSIs that target the LBD [34, 35, 36]. Hence, compounds that can simultaneously target AR‐FL and AR‐V7 may provide novel strategies to overcome the resistance of cells to second‐generation ARSIs. In the present study, we report that TAS3681 is a novel small‐molecule compound that inhibits both AR‐FL and constitutively active AR‐V7 and overcomes resistance to enzalutamide therapy in CRPC in *in vitro* and *in vivo* models.

AR‐V7 is rarely expressed in primary PCa but its expression is often observed following ADT, with further increase in expression following treatment with ARSIs [37]. These findings were consistent with the observations using the experimental models. Expression of truncated AR‐Vs can be significantly upregulated after treatment with abiraterone in CRPC xenografts and has been observed in PCa cells after long‐term treatment with enzalutamide [38, 39]. In this study, we used SAS MDV No. 3‐14 cells established from LNCaP xenograft tumors in castrated mice under continuous enzalutamide treatment. SAS MDV No. 3‐14 cells express AR‐Vs including AR‐V7. Endogenous AR‐FL and AR‐V7 function as proliferative drivers in SAS MDV No. 3‐14 cells [8]. In contrast to enzalutamide, TAS3681 effectively and dose‐dependently suppressed the growth of SAS MDV No. 3‐14 cells and reduced the protein levels of AR‐FL and AR‐Vs including AR‐V7 *in vitro* and *in vivo*. Additionally, TAS3681 reduced both AR‐FL and AR‐Vs protein levels in the nucleus, where they function as transcription factors. TAS3681 reduced the expression of AR‐FL‐regulated genes such as *PSA* and, more importantly, that of AR‐V7‐regulated genes, such as *UBE2C* and *CDC20*, whereas enzalutamide did not affect gene expression. Recently, several compounds have been found to affect AR‐Vs. EPI‐7386 and UT‐155 are inhibitors of the N‐terminal domain of AR and block AR‐V7 activity [40, 41]. An FDA‐approved anthelminthic drug, niclosamide [42], was previously identified as a downregulator of AR‐V7. These compounds exhibit a synergistic effect with enzalutamide and resensitize treatment‐resistant PCa cells to enzalutamide. Therefore, combining second‐generation ARSIs with AR‐V7 inhibitors could be an attractive strategy to overcome ARSI resistance in PCa. TAS3681 is a new class of second‐generation ARSIs that can simultaneously target both AR‐FL and AR‐V7. Therefore, TAS3681, as a mono‐therapy, could potentially overcome enzalutamide resistance. Drug–drug interactions could also be avoided, along with any other adverse effects.

Recent analyses of cell‐free circulating tumor DNA (ctDNA) from patients with mCRPC have shown that *AR* amplification is associated with resistance to second‐generation ARSIs [24, 26, 43, 44]. Consistent with these findings, increased expression of AR‐FL in PCa cells after treatment with ARSIs, including abiraterone and enzalutamide, has been reported in *in vitro* and *in vivo* models [39, 45]. For instance, two enzalutamide‐resistant sub‐lines (LAPC4 and DuCaP cells) established by long‐term enzalutamide exposure exhibited overexpressed AR‐FL and decreased sensitivity to enzalutamide [37]. In the present study, we used VCaP cells transfected with an AR‐expressing vector as the AR‐overexpressing PCa cell model. VCaP cells exhibit endogenous *AR* amplification, but second‐generation ARSIs, including enzalutamide and darolutamide, are effective against the cells [46]. This finding was reproduced in our study, and enzalutamide completely blocked DHT‐induced AR transcriptional activation and proliferation in VCaP cells. The ineffectiveness of enzalutamide in AR‐overexpressing VCaP cells by transfection with an AR‐expressing vector suggests that an increase in AR expression is a cause of enzalutamide resistance. TAS3681 uniquely reduced AR protein levels in cells and effectively blocked DHT‐induced AR transcriptional activation and cell proliferation in both parental and AR‐overexpressing cells. These results showed that TAS3681 is fully antagonistic to AR overexpression, probably through its AR‐reducing effect and AR pure antagonism. Further studies with different AR‐overexpressing models are necessary to consider the notable effects of TAS3681 in the future.

Androgen receptor mutations in the LBD in response to ARSIs are a well‐known mechanism underlying therapeutic resistance. Although rarely observed in non‐metastatic castration‐sensitive patients [36], AR‐LBD mutations are frequently detected in heavily pretreated mCRPC patients, suggesting that they are advantageous to clonal propagation [47]. TAS3681 is a pure antagonist of all the studied AR mutations that can lead to resistance to both first‐ and second‐generation ARSIs. In contrast to apalutamide and enzalutamide, TAS3681 antagonized the AR F877L mutation and suppressed the proliferation of F877L AR cells. Moreover, TAS3681 functioned as an antagonist of the double‐mutant ARs F877L/T878A and H875Y/T878A, conferring the activation of AR‐mediated transcription by enzalutamide and apalutamide. Recently, it was reported that the AR F877L mutation leads to enzalutamide and apalutamide resistance, as it converts them into agonists [7, 48]. Furthermore, enzalutamide acts as a weak partial agonist for AR F877L and a strong partial agonist for double‐mutant ARs F877L/T878A and H875Y/T878A [21, 48]. In the present study, darolutamide exhibited agonistic effects on the mutated ARs V716M [49] and H875Y, as reported, whereas no significant agonist profile was observed with TAS3681. Both mutations have been identified in patients with CRPC, and V716M was identified in ctDNA collected at the time of resistance to abiraterone and bicalutamide [50]. Therefore, TAS3681 functions as an AR pure antagonist against various clinically relevant AR mutations that are generated in response to treatment with antiandrogen. Our preclinical data support further evaluation of the clinical responses to TAS3681 in current second‐generation ARSI‐resistant CRPC patients.

The balance between the rates of synthesis and proteasomal degradation of AR protein determines the steady‐state levels of AR protein in the cells [45]. For example, HSP90 inhibitors increase the rate of degradation of AR protein by disrupting HSP90‐AR interaction that is essential for AR stability [51]. In this study, TAS3681 did not increase the rate of AR degradation in the presence of CHX, suggesting that the mechanism of AR protein level reduction by TAS3681 does not involve enhanced protein degradation. In another experiment, TAS3681 reduced AR protein, but not *AR* mRNA levels, in the presence of Act D; moreover, TAS3681 did not show a significant effect on the transcription of AR. These findings suggest that TAS3681 reduced the rate of AR protein synthesis. A non‐selective protein synthesis inhibitor, CHX, reduced both AR and GAPDH levels in the presence of Act D; however, TAS3681 did not promote the reduction of GAPDH under the same conditions, indicating that these two compounds downregulate AR protein by different mechanisms. The results of our studies also suggest that TAS3681 downregulates both AR‐FL and AR‐V7 protein levels at the translational level. The co‐expression of AR‐FL and AR‐V7 in the same cells has been explained by selective alternative AR splicing [44]. Several splicing factors have been shown to selectively regulate AR‐V7 splicing without a significant impact on AR‐FL splicing in response to ADT [52]. A second‐generation HSP90 inhibitor blocks mRNA splicing of AR‐V7 but not FL‐AR, and reduces AR‐V7 mRNA and protein levels [53]. The alternative AR splicing is a major mechanism that affects the expression of FL‐AR and AR‐V7, but TAS3681 may not have an effect on it, because our results suggest that TAS3681 does not affect the mRNA expression levels of FL‐AR and AR‐V7. AR‐FL and AR‐V7 protein levels are also regulated by mRNA translation initiation, including cap‐dependent translational machinery [42]. The potential mechanism should be the focus of future studies to elucidate the unique mechanism of action of TAS3681.

This study has some limitations. Unfortunately, the *in vitro* PCa cell models mimicking the ARSI resistance often observed in clinical mCRPC were limited to SAS MDV No. 3‐14 cells and VCaP cells in our study. Our results will be further validated as soon as new cell lines become available. Patient‐derived xenograft models of abiraterone‐ and enzalutamide‐resistant PCa could be a solution to these limitations [54].

## 5 Conclusion

In summary, our experimental data indicate that TAS3681 is part of the next‐generation ARSIs, which antagonize various AR mutants that can lead to resistance to first‐ and second‐generation ARSIs. TAS3681 also has a unique AR‐FL and AR‐Vs downregulation activity, which is accompanied by an antitumor effect in AR‐V7 expressing enzalutamide‐resistant cells *in vitro* and *in vivo*. These biological profiles support TAS3681 as a potential therapeutic candidate against multiple resistance mechanisms of the AR pathway in patients with mCRPC who have progressed to treatment with second‐generation ARSIs. This compound is currently under clinical investigation in a phase I study of patients with mCRPC previously treated with abiraterone acetate and/or enzalutamide (NCT02566772).

## Conflict of interest

All authors are employed at Taiho Pharmaceutical Co., Ltd.

## Author contributions

KM conceptualized and designed the study. DK, MS, HM, RF, and KY performed *in vivo* experiments. SY, DK, MS, MT, YT, HM, SO, and MA performed *in vitro* experiments. SY, DK, MS, MT, YT, HM, RF, and KY analyzed the data. KM and SY supervised the study. SY wrote the manuscript. All authors participated in data interpretation, manuscript review and editing. All authors read and approved the final manuscript.

### Peer review

The peer review history for this article is available at https://www.webofscience.com/api/gateway/wos/peer‐review/10.1002/1878‐0261.13641.

## Supporting information


**Fig. S1.** Effect of TAS3681, enzalutamide, and bicalutamide on the transcriptional activity of the wild‐type AR in VCaP cells.
**Fig. S2.** Cell proliferation assay of TAS3681 in AR‐negative human cancer cells.
**Fig. S3.** Cell proliferation assay of TAS3681in the presence of DHT in AR‐negative human cancer cells.
**Fig. S4.** Effect of TAS3681, enzalutamide, and TOK‐001 on AR and GAPDH protein expression in VCaP cells.
**Fig. S5.** Expression of AR, ERα, GR, PR‐A, PR‐B, and GAPDH protein in MCF‐7 and T‐47D cells after treatment with TAS3681.
**Fig. S6.** Effect of antiandrogens on AR subcellular localization in the absence of androgen.
**Fig. S7.** Antagonist activities of hydroxyflutamide, bicalutamide, enzalutamide, and TAS3681 against wild‐type or mutant ARs.
**Fig. S8.** Effect of TAS3681, enzalutamide, apalutamide, and darolutamide on the transcriptional activity of mutated and wild‐type AR.
**Fig. S9.** Downregulation of AR‐V7 protein expression in SAS MDV No. 3‐14 cells treated with TAS3681.
**Fig. S10.** Downregulation of AR protein expression in SAS MDV No. 3‐14 cells treated with TAS3681.
**Fig. S11.** Effect of TAS3681, enzalutamide, and bicalutamide on AR‐FL, AR‐V, and GAPDH protein expression in 22Rv1 cells.
**Fig. S12.** Effect of TAS3681 on AR‐V7‐related target genes in SAS MDV No. 3‐14 cells.
**Fig. S13.** Effect of TAS3681 and 17‐AAG on the stability of AR and β‐actin proteins in LNCaP cells.
**Fig. S14.** Effect of TAS3681 and CHX on AR and GAPDH protein expression in LNCaP cells in the presence of Act D.
**Fig. S15.** Effect of TAS3681, enzalutamide, and actinomycin D on *AR* mRNA expression in LNCaP cells.
**Fig. S16.** TAS3681 downregulates AR‐Vs protein levels at the translational level.
**Fig. S17.** Effect of TAS3681, enzalutamide on *AR‐V7* mRNA expression in SAS MDV No.3‐14 cells.
**Fig. S18.** Changes in body weight during TAS3681 treatment in castrated SCID mice implanted with human prostate cancer SAS MDV No.3‐14 cells.
**Fig. S19.** Effect of TAS3681 on serum prostate‐specific antigen (PSA) levels in castrated SCID mice bearing SAS MDV No. 3‐14 human prostate cancer xenografts.
**Fig. S20.** Downregulation of AR‐FL (left) and AR‐Vs protein (right) expression in tumors of castrated SCID mice after treatment with TAS3681.
**Table S1.** qPCR primers.
**Table S2.** Inhibition of [^3^H]methyltrienolone binding to wild‐type AR and T878Amutant AR by TAS3681.
**Table S3.** Effects of TAS3681 on AR wild‐type and T878A mutants in cell‐based transactivation assays.
**Table S4.** Effects of TAS3681 on DHT‐induced proliferation of VCaP (AR wild type) and LNCaP (T878A mutants).
**Table S5.** Inhibition of AR nuclear translocation in U‐2 OS tomato‐AR cells.
**Table S6.** IC_50_ values of AR antagonist against mutant and wild‐type AR activation by DHT.

## Data Availability

All relevant data are within the paper and its Supporting Information files.
