# Supplementary material for: TAS3681, an androgen receptor antagonist, prevents drug resistance driven by aberrant androgen receptor signaling in prostate cancer
Source: Mol Oncol. 2024 Apr 10;18(8):1980–2000. doi: 10.1002/1878-0261.13641 (PMC11306513; doi:10.1002/1878-0261.13641)
Supplement: Supplementary file 1 — Fig. S1. Effect of TAS3681, enzalutamide, and bicalutamide on the transcriptional activity of the wild‐type AR in VCaP cells. Fig. S2. Cell proliferation assay of TAS3681 in AR‐negative human cancer cells. Fig. S3. Cell proliferation assay of TAS3681in the presence of DHT in AR‐negative human cancer cells. Fig. S4. Effect of TAS3681, enzalutamide, and TOK‐001 on AR and GAPDH protein expression in VCaP cells. Fig. S5. Expression of AR, ERα, GR, PR‐A, PR‐B, and GAPDH protein in MCF‐7 and T‐47D cells after treatment with TAS3681. Fig. S6. Effect of antiandrogens on AR subcellular localization in the absence of androgen. Fig. S7. Antagonist activities of hydroxyflutamide, bicalutamide, enzalutamide, and TAS3681 against wild‐type or mutant ARs. Fig. S8. Effect of TAS3681, enzalutamide, apalutamide, and darolutamide on the transcriptional activity of mutated and wild‐type AR. Fig. S9. Downregulation of AR‐V7 protein expression in SAS MDV No. 3‐14 cells treated with TAS3681. Fig. S10. Downregulation of AR protein expression in SAS MDV No. 3‐14 cells treated with TAS3681. Fig. S11. Effect of TAS3681, enzalutamide, and bicalutamide on AR‐FL, AR‐V, and GAPDH protein expression in 22Rv1 cells. Fig. S12. Effect of TAS3681 on AR‐V7‐related target genes in SAS MDV No. 3‐14 cells. Fig. S13. Effect of TAS3681 and 17‐AAG on the stability of AR and β‐actin proteins in LNCaP cells. Fig. S14. Effect of TAS3681 and CHX on AR and GAPDH protein expression in LNCaP cells in the presence of Act D. Fig. S15. Effect of TAS3681, enzalutamide, and actinomycin D on AR mRNA expression in LNCaP cells. Fig. S16. TAS3681 downregulates AR‐Vs protein levels at the translational level. Fig. S17. Effect of TAS3681, enzalutamide on AR‐V7 mRNA expression in SAS MDV No.3‐14 cells. Fig. S18. Changes in body weight during TAS3681 treatment in castrated SCID mice implanted with human prostate cancer SAS MDV No.3‐14 cells. Fig. S19. Effect of TAS3681 on serum prostate‐specific antigen (PSA) levels in castrated [file MOL2-18-1980-s001.zip › mol213641-sup-0001-Supinfo.pdf]

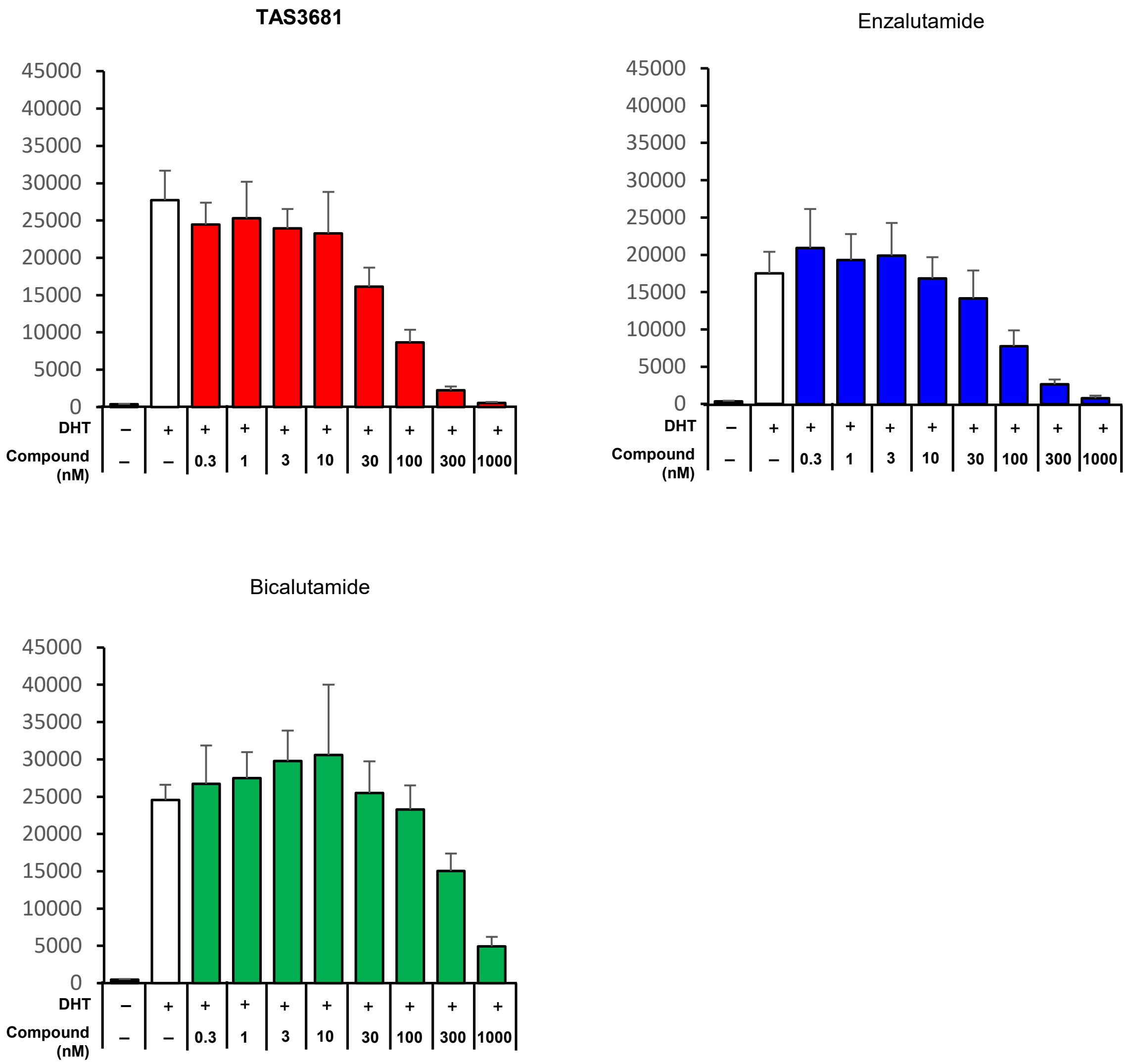

Supplementary Fig. S1

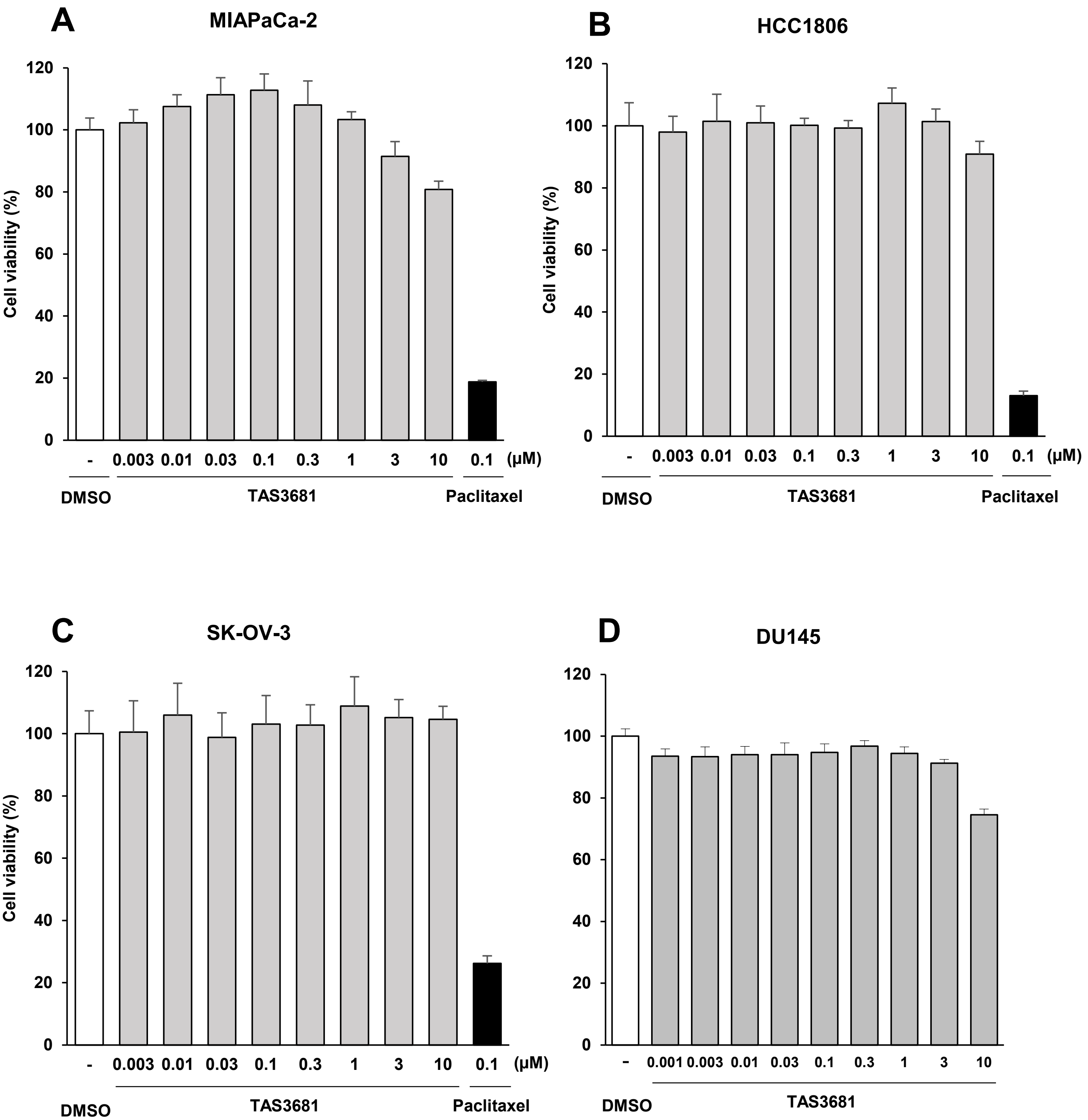

Supplementary Fig. S2

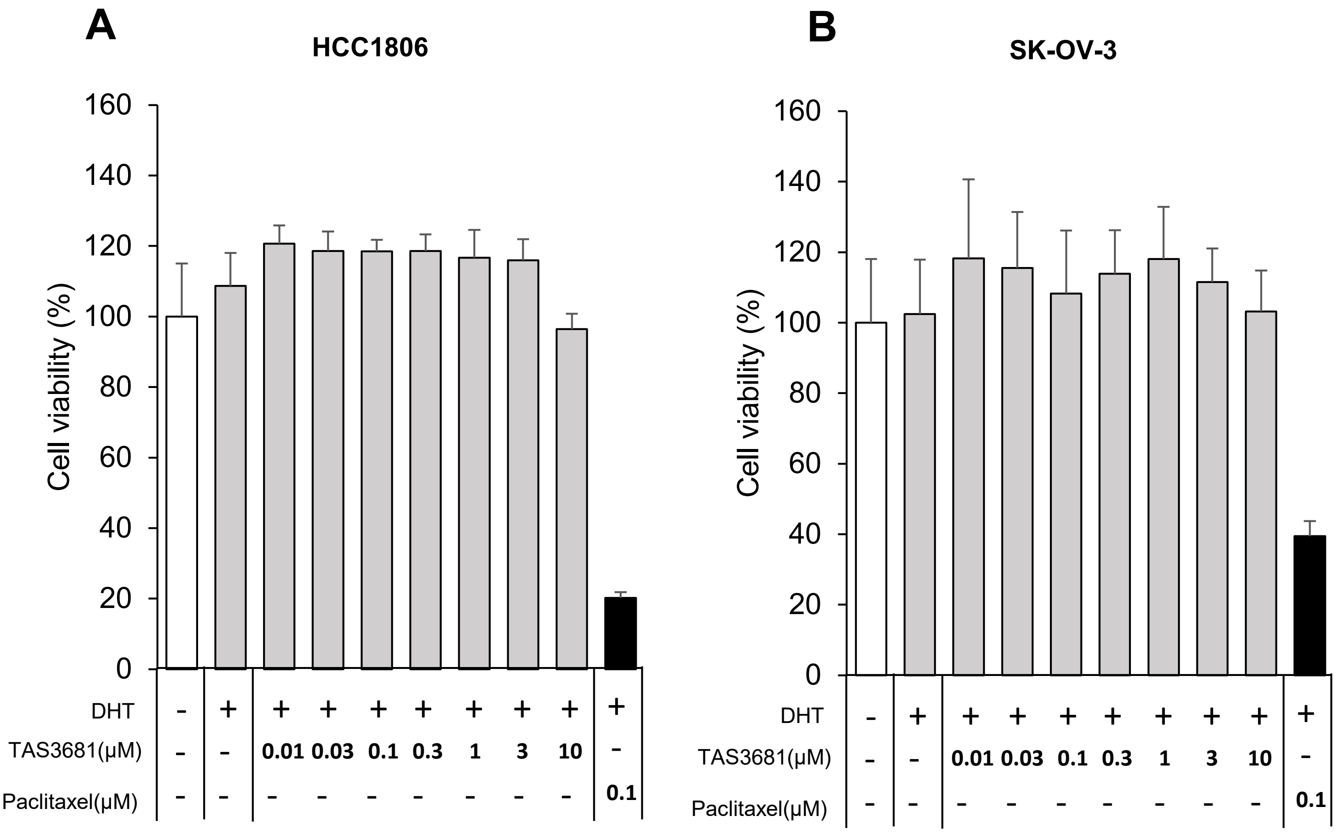

Supplementary Fig. S3

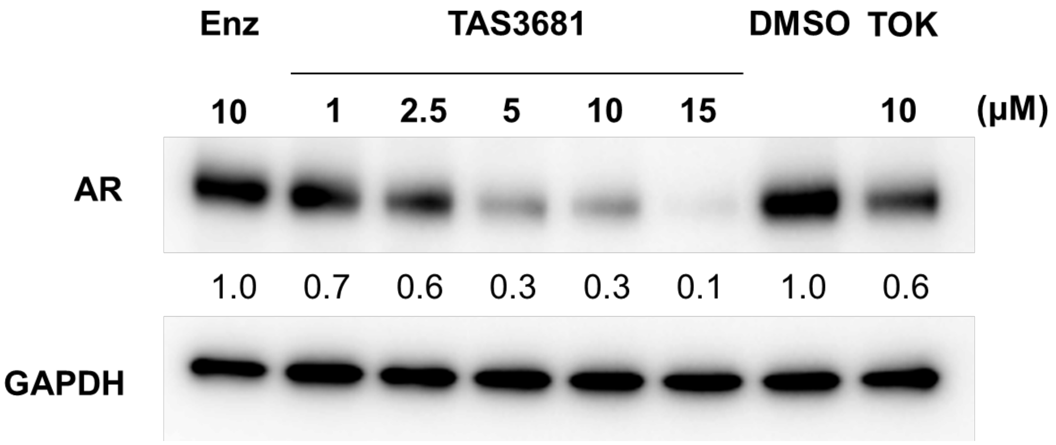

Supplementary Fig. S4

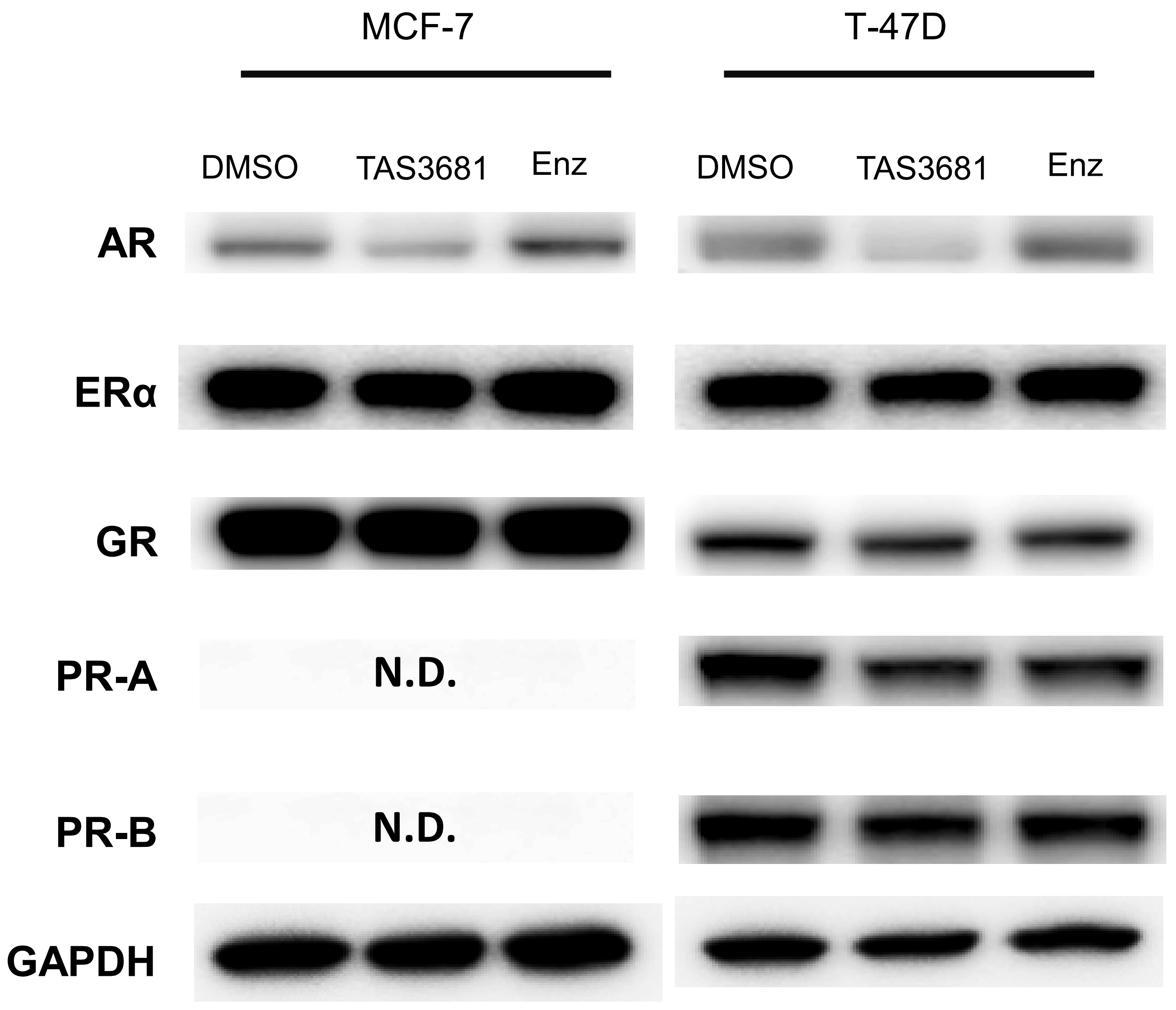

Supplementary Fig. S5

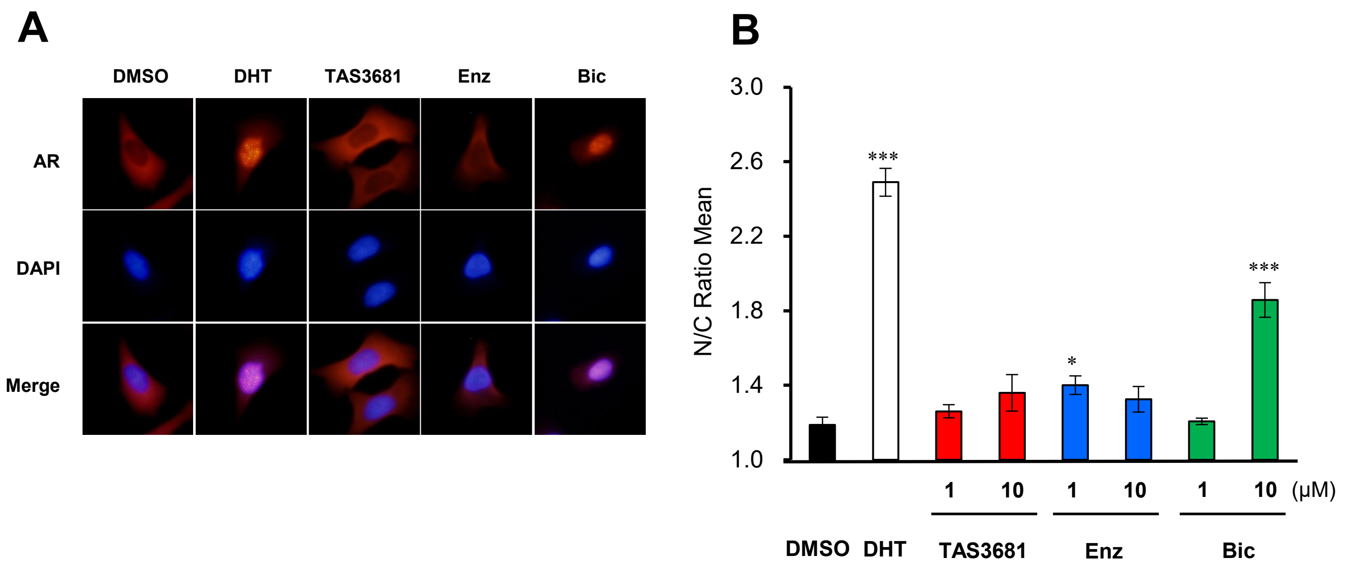

Supplementary Fig. S6

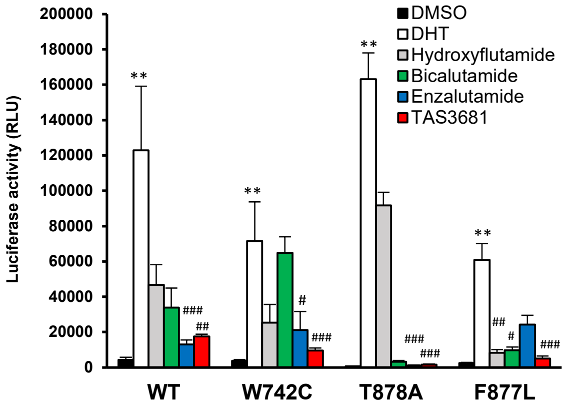

Supplementary Fig. S7

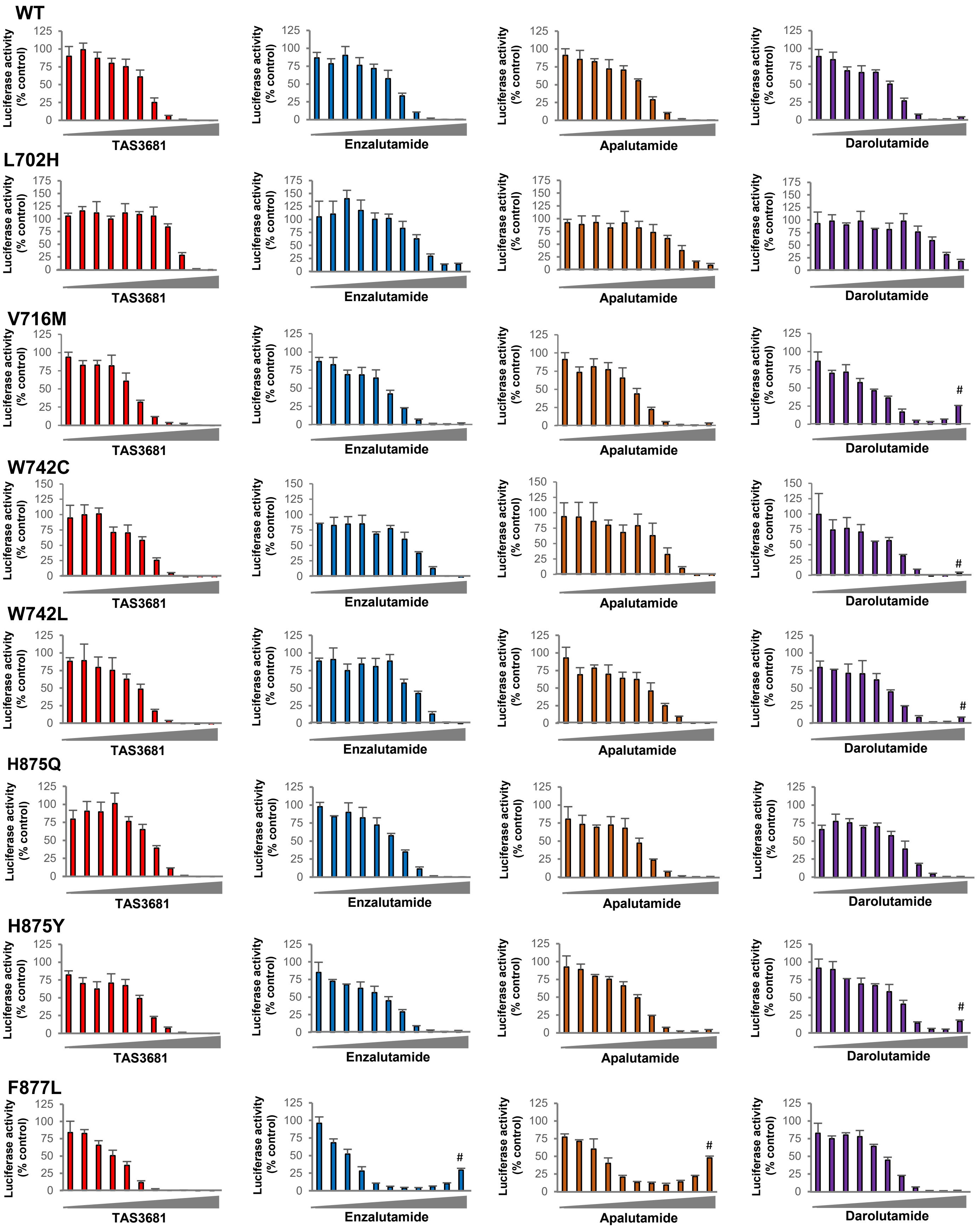

Supplementary Fig. S8.

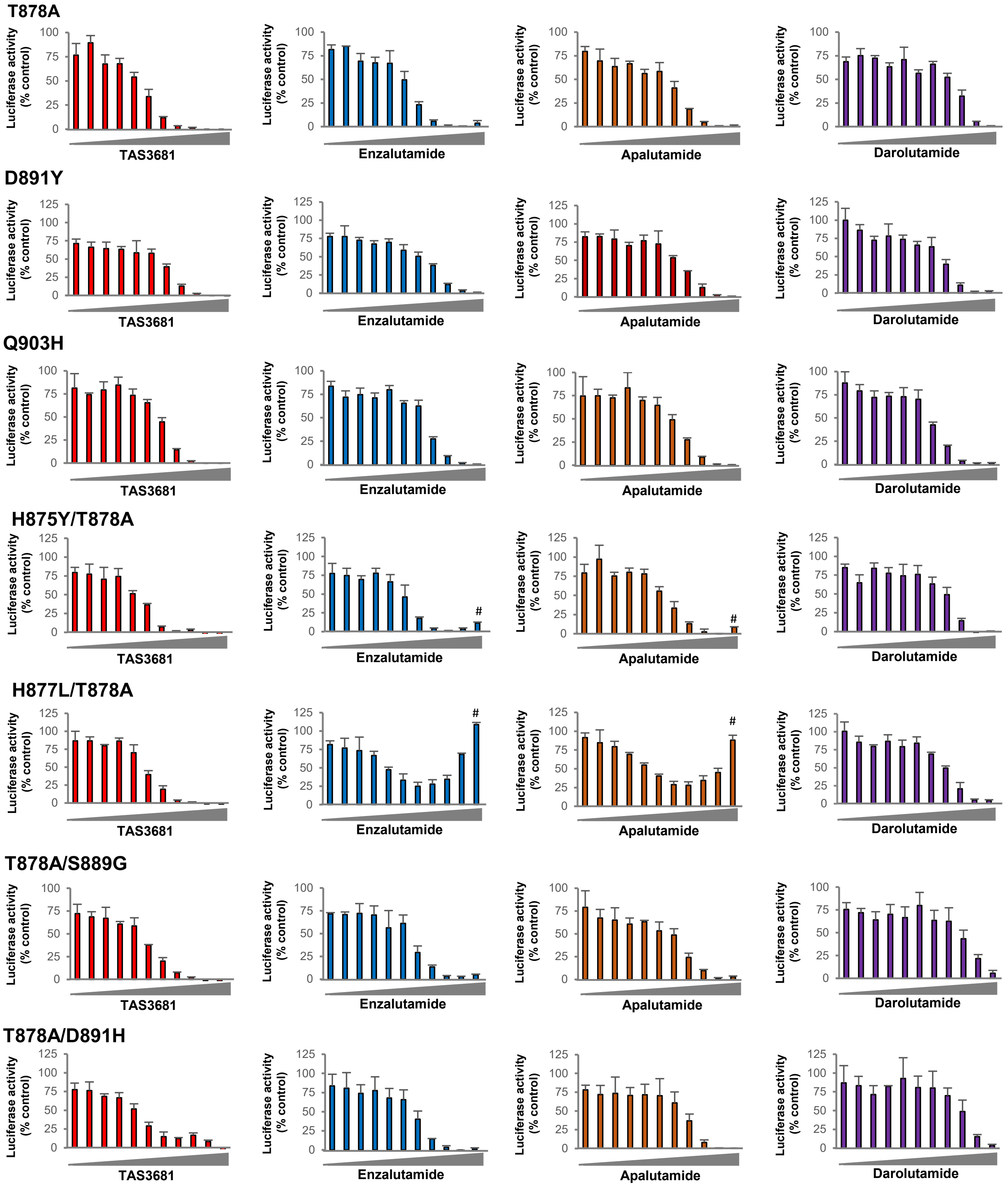

Supplementary Fig. S8 (continued)

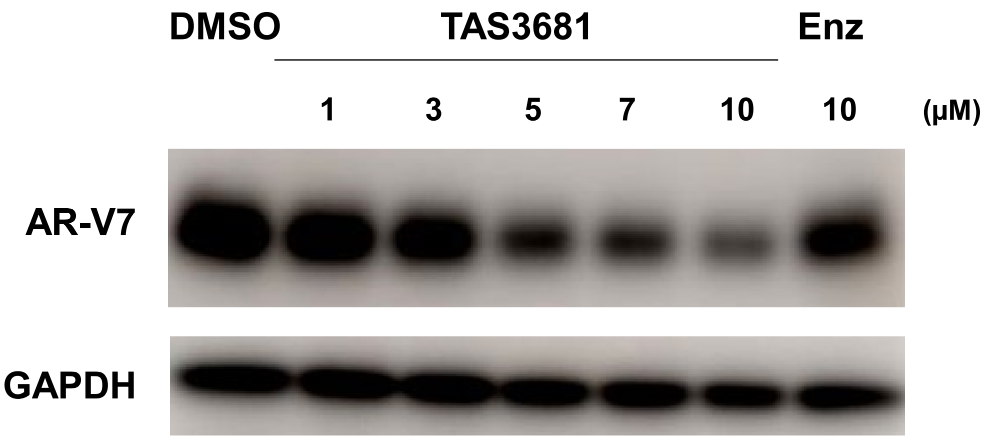

Supplementary Fig. S9

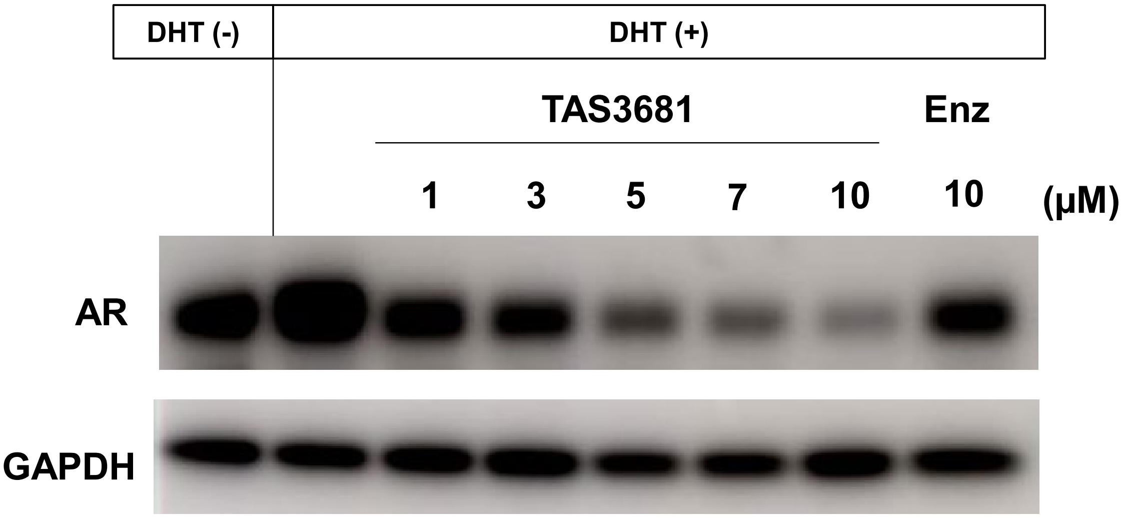

Supplementary Fig. S10

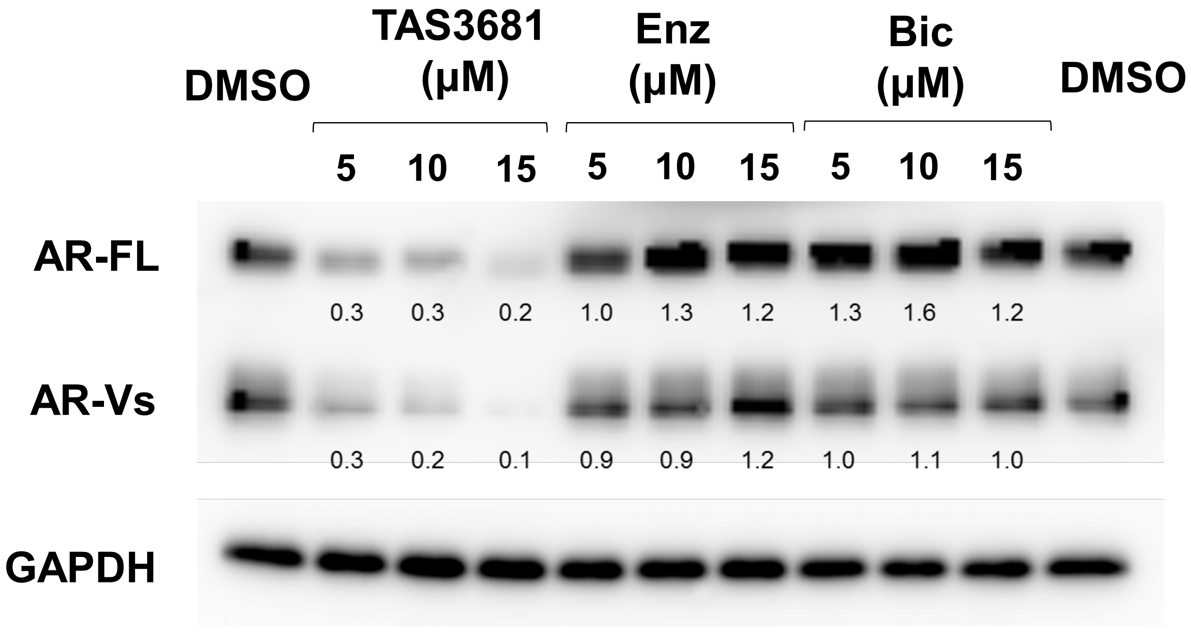

Supplementary Fig. S11

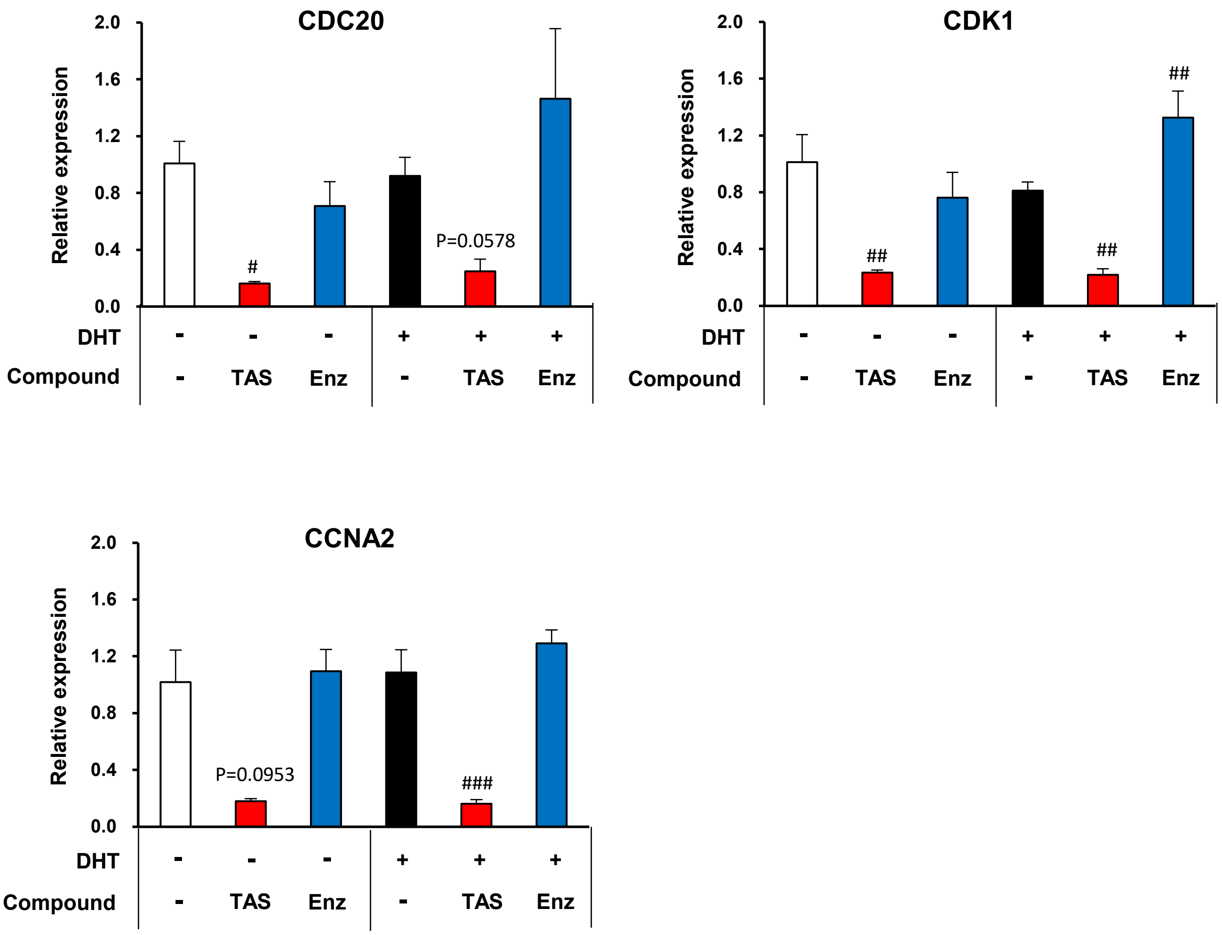

Supplementary Fig. S12

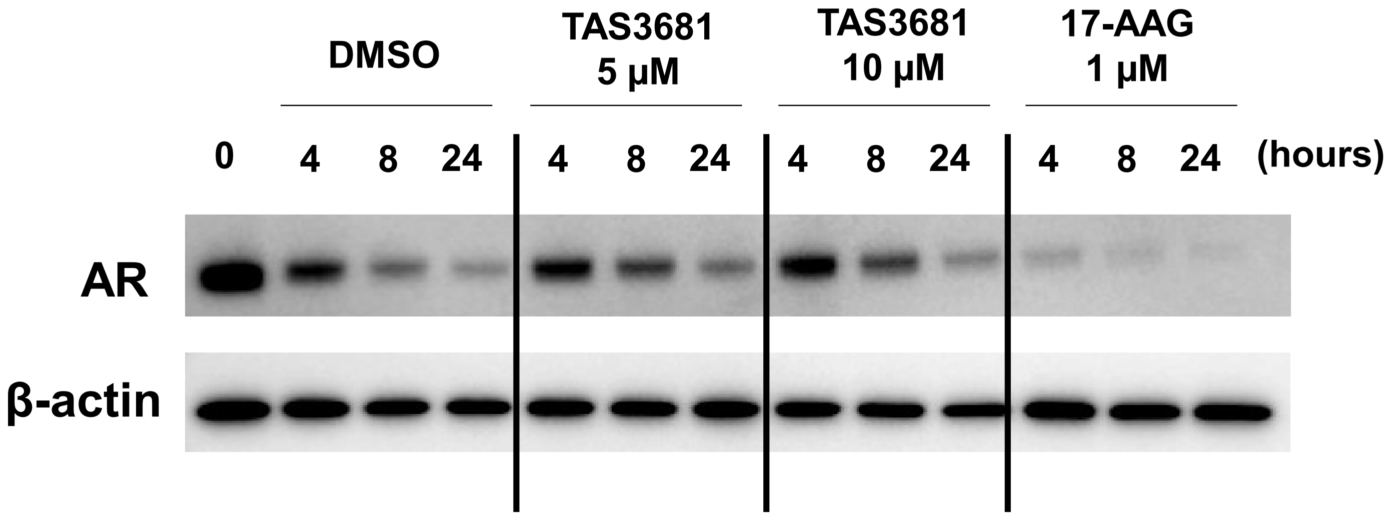

Supplementary Fig. S13

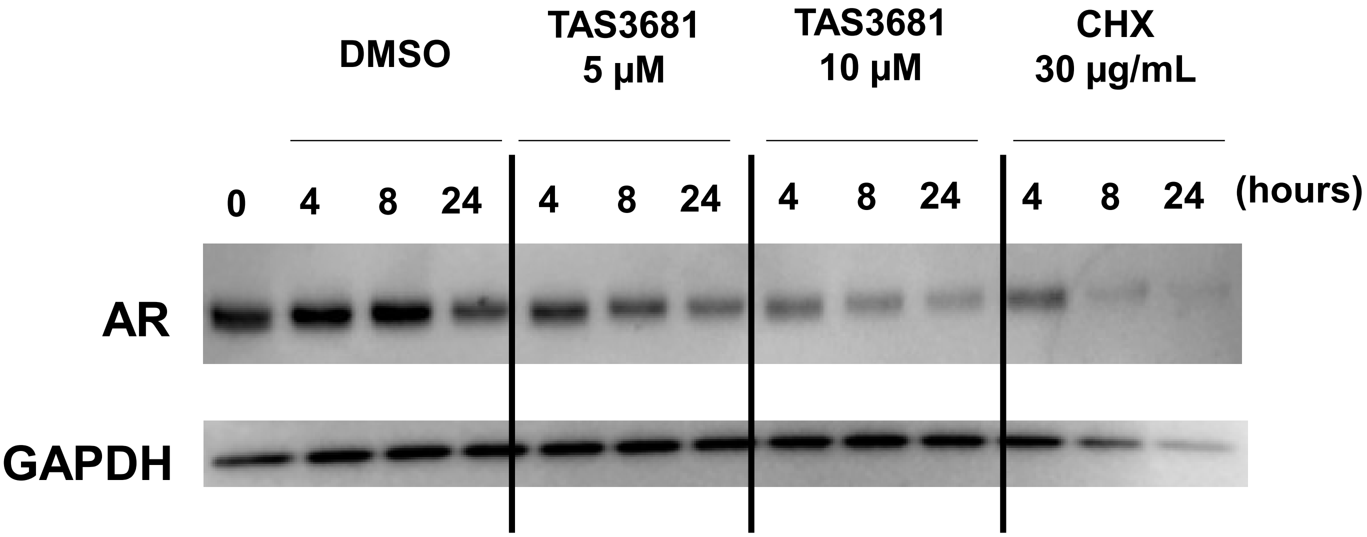

Supplementary Fig. S14

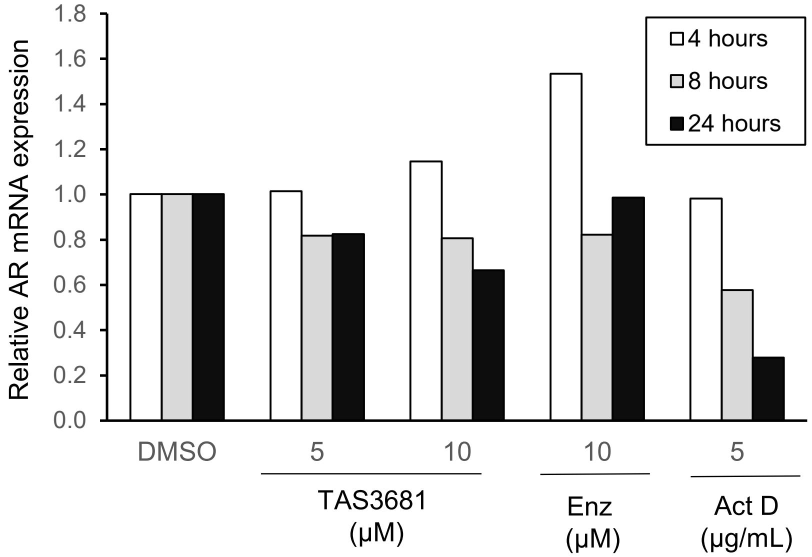

Supplementary Fig. S15

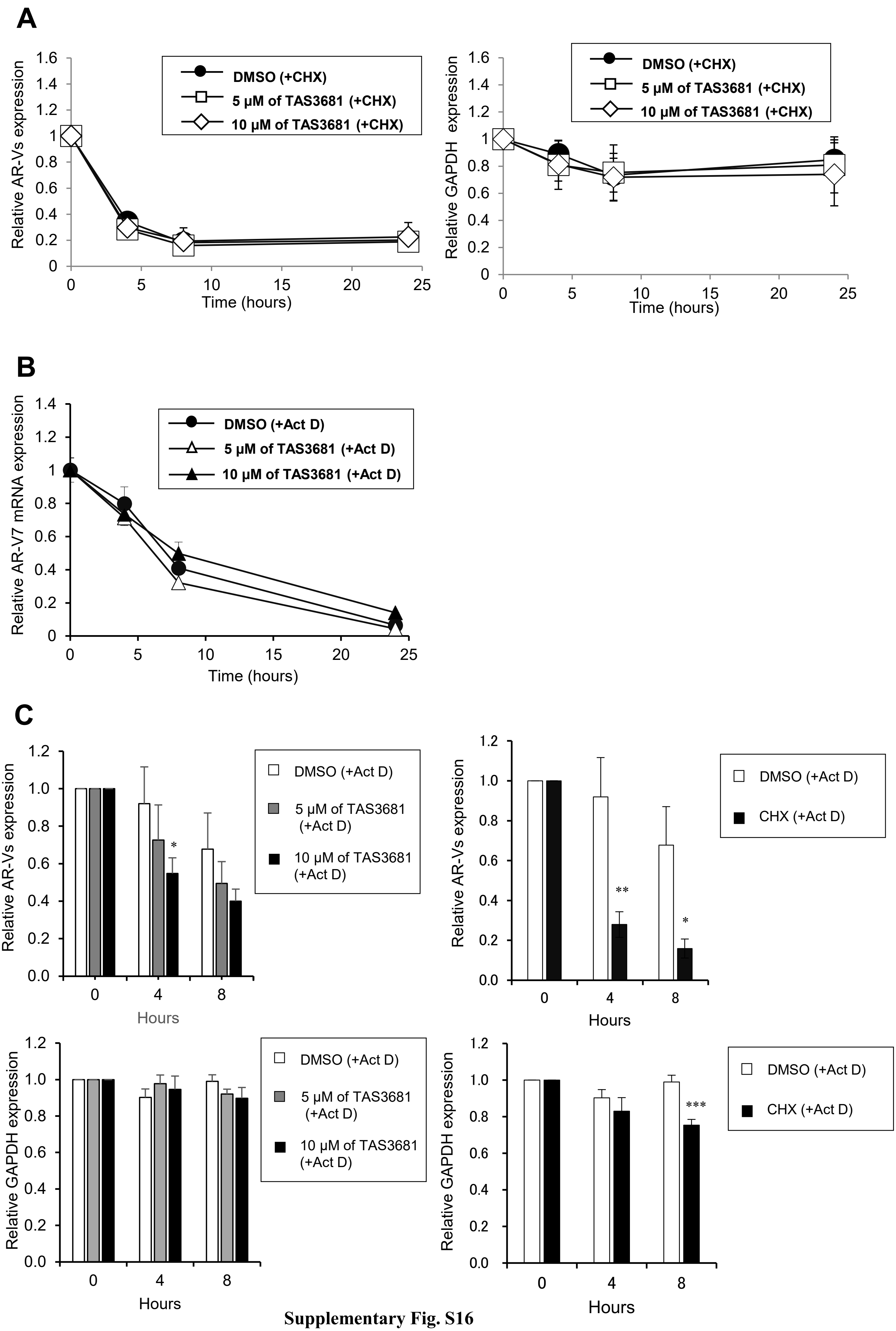

Supplementary Fig. S16

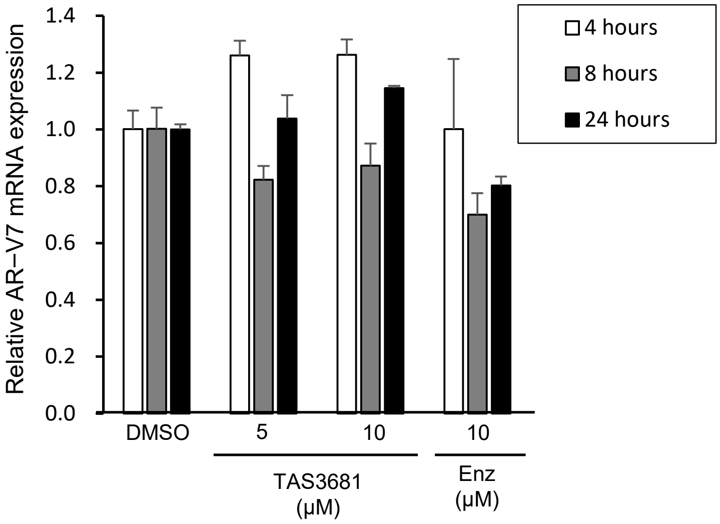

Supplementary Fig. S17

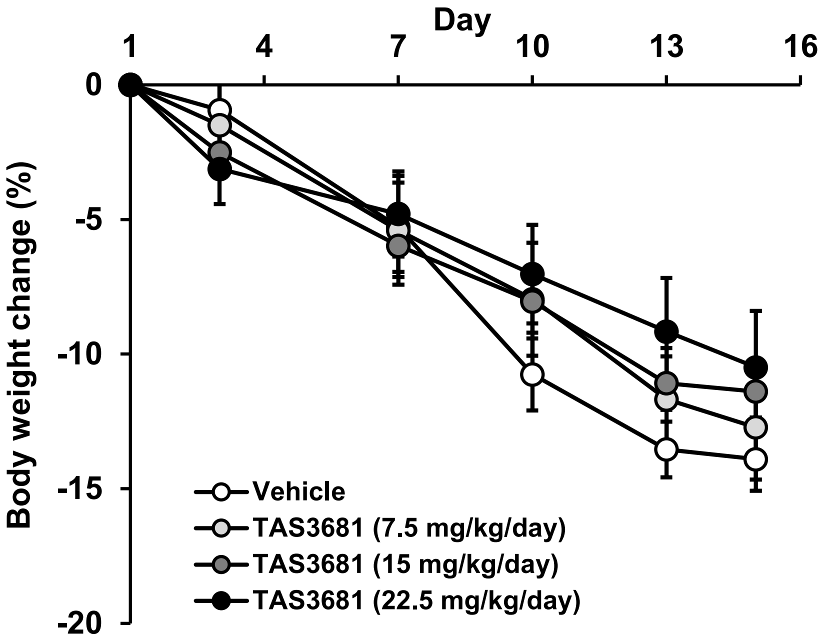

Supplementary Fig. S18

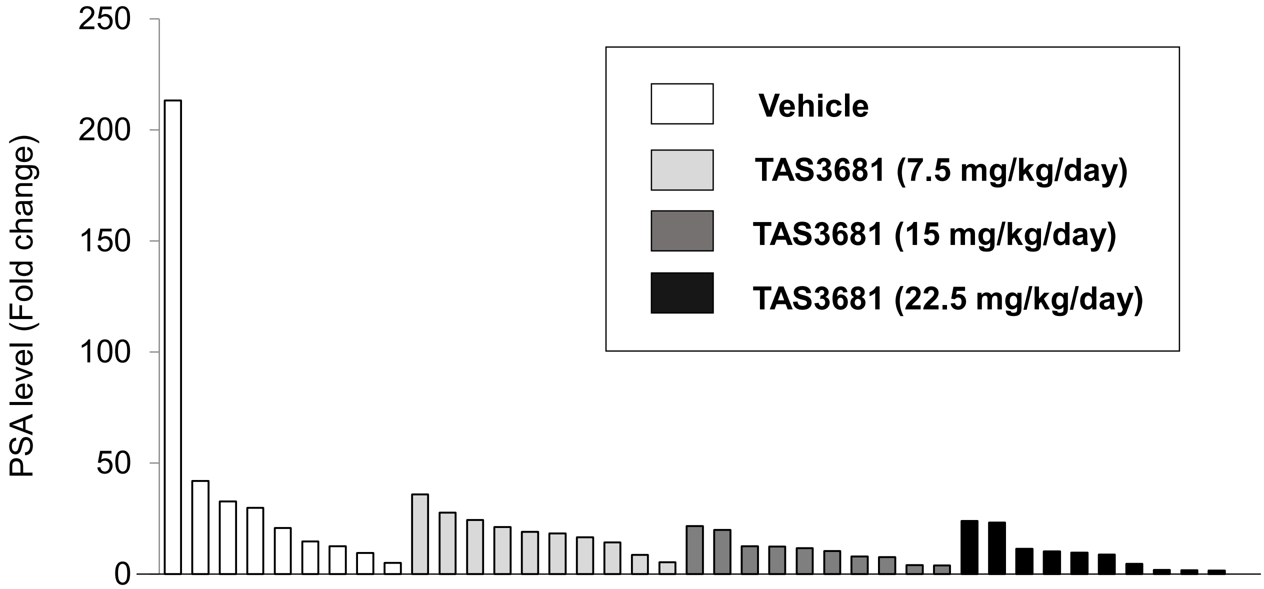

Supplementary Fig. S19

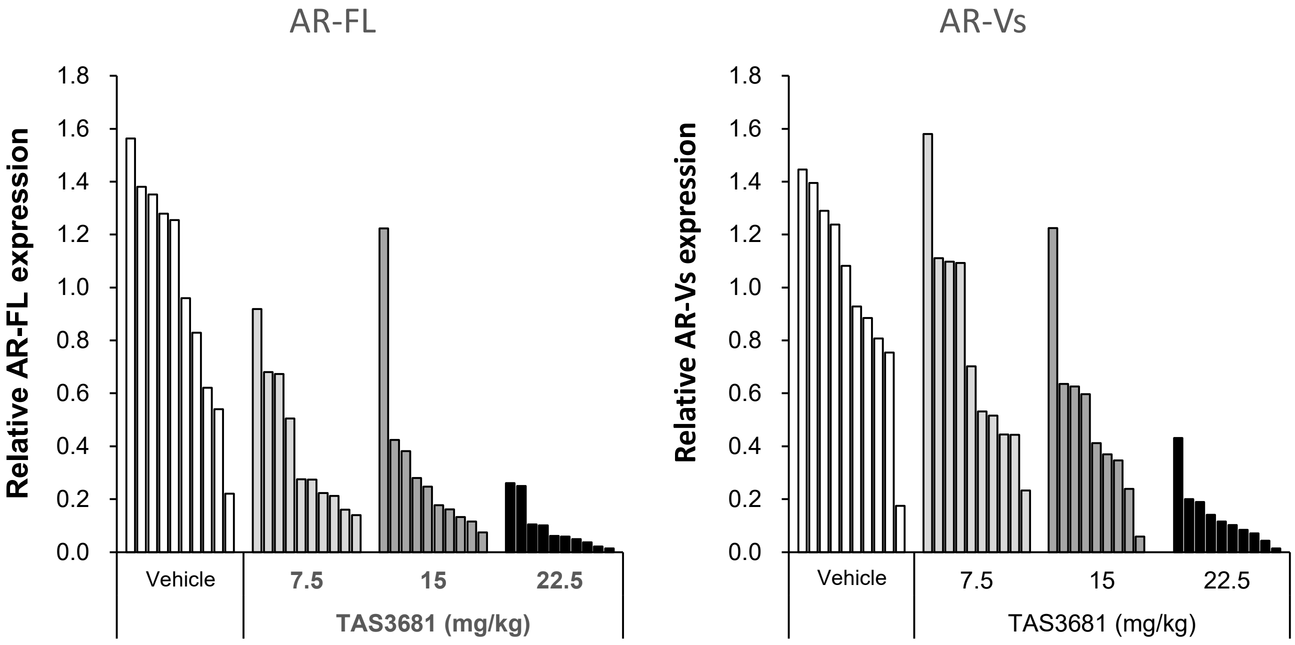

Supplementary Fig. S20

Supplementary Table S1.

ChIP qPCR primer		
**Gene**	**Direction**	**Sequence (5' to 3')**
**KLK3**	Forward	TGGGACAACTTGCAAACCTG
	Reverse	CCAGAGTAGGTCTGTTTTCAATCCA
**TMPRSS2**	Forward	TGGTCCTGGATGATAAAAAAAGTTT
	Reverse	GACATACGCCCCACAACAGA

qPCR primer	
**Gene**	**Catalog number**
**AR**	Hs00171172_m1<br>Hs0462016_m1
**CDC20**	Hs00426680_mH
**CCNA2**	Hs00996788_m1
**UBE2C**	Hs00964100_g1
**CDK1**	Hs00938777_m1
**GAPDH**	4310884E<br>4310859<br>Hs99999905_m1

Supplementary Table S2.

Compounds	Wild-type AR		T878A mutant AR	
	IC_{50}	Ki	IC_{50}	Ki
TAS3681	15.5 nM	7.39 nM	40.6 nM	23.8 nM
Enzalutamide	14.8 nM	7.11 nM	38.5 nM	22.6 nM
Apalutamide	12.3 nM	6.01 nM	195 nM	115 nM

- VCaP cell lysate for wild-type AR or LNCaP cell lysate for T878A mutant AR were incubated for 20 hours at 4°C with [3H]methyltrienolone and increasing concentrations of TAS3681, enzalutamide, and apalutamide.
- The 50% inhibition concentration (IC_{50}) values were determined using Prism software (GraphPad Software, Inc., CA, USA).

Supplementary Table S3.

Compounds	IC_{50} values		
	Wild-type AR		T878A mutant AR
	COS-7/wt-AR	VCaP	LNCaP
TAS3681	52.7 nM	60.9 nM	10.1 nM
Enzalutamide	59.7 nM	117.7 nM	12.5 nM
Bicalutamide	429 nM	662.5 nM	60.8 nM

Supplementary Table S4.

Compounds	IC_{50} values	
	VCaP	LNCaP
TAS3681	170 nM	18 nM
Enzalutamide	180 nM	55 nM
Bicalutamide	490 nM	340 nM

Supplementary Table S5.

Compounds	IC_{50} values
TAS3681	63.7 nM
Enzalutamide	103 nM
Bicalutamide	> 1000 nM

Supplementary Table S6.

	DHT (nM)	IC$_{50}$ (μM)			
		TAS3681	Enzalutamide	Apalutamide	Darolutamide
WT	0.1	0.099	0.088	0.071	0.044
L702H	1	1.985	1.522	1.232	4.126
V716M	0.1	0.039	0.035	0.048	0.017[a]
W742C	0.1	0.084	0.284	0.289	0.048[a]
W742L	1	0.048	0.431	0.087	0.033[a]
H875Q	0.1	0.158	0.104	0.037	0.058
H875Y	0.1	0.030	0.026	0.058	0.083[a]
F877L	0.1	0.008	0.003[a]	0.005[a]	0.045
T878A	0.1	0.022	0.041	0.037	0.172
D891Y	0.1	0.027	0.102	0.195	0.240
Q903H	0.1	0.122	0.158	0.118	0.109
H875Y/T878A	0.1	0.022	0.037[a]	0.098[a]	0.303
F877L/T878A	0.1	0.058	N.D.[a]	N.D.[a]	0.628
T878A/S889G	0.1	0.017	0.038	0.042	0.516
T878A/D891H	0.1	0.019	0.091	0.147	1.778

IC$_{50}$ values were estimated from luciferase activity (% control) using XLfit® version 5.5.0.5 (ID Business Solutions, Guildford, UK) .
[a] More than 5% increase in relative luciferase activity (% control) was observed at 30 μM compared with that at 10 μM.
N.D.: not determined
