# Supplementary material for: TAS3681, an androgen receptor antagonist, prevents drug resistance driven by aberrant androgen receptor signaling in prostate cancer
Source: Mol Oncol. 2024 Apr 10;18(8):1980–2000. doi: 10.1002/1878-0261.13641 (PMC11306513; doi:10.1002/1878-0261.13641)
Supplement: Supplementary file 1 — Fig. S1. Effect of TAS3681, enzalutamide, and bicalutamide on the transcriptional activity of the wild‐type AR in VCaP cells. Fig. S2. Cell proliferation assay of TAS3681 in AR‐negative human cancer cells. Fig. S3. Cell proliferation assay of TAS3681in the presence of DHT in AR‐negative human cancer cells. Fig. S4. Effect of TAS3681, enzalutamide, and TOK‐001 on AR and GAPDH protein expression in VCaP cells. Fig. S5. Expression of AR, ERα, GR, PR‐A, PR‐B, and GAPDH protein in MCF‐7 and T‐47D cells after treatment with TAS3681. Fig. S6. Effect of antiandrogens on AR subcellular localization in the absence of androgen. Fig. S7. Antagonist activities of hydroxyflutamide, bicalutamide, enzalutamide, and TAS3681 against wild‐type or mutant ARs. Fig. S8. Effect of TAS3681, enzalutamide, apalutamide, and darolutamide on the transcriptional activity of mutated and wild‐type AR. Fig. S9. Downregulation of AR‐V7 protein expression in SAS MDV No. 3‐14 cells treated with TAS3681. Fig. S10. Downregulation of AR protein expression in SAS MDV No. 3‐14 cells treated with TAS3681. Fig. S11. Effect of TAS3681, enzalutamide, and bicalutamide on AR‐FL, AR‐V, and GAPDH protein expression in 22Rv1 cells. Fig. S12. Effect of TAS3681 on AR‐V7‐related target genes in SAS MDV No. 3‐14 cells. Fig. S13. Effect of TAS3681 and 17‐AAG on the stability of AR and β‐actin proteins in LNCaP cells. Fig. S14. Effect of TAS3681 and CHX on AR and GAPDH protein expression in LNCaP cells in the presence of Act D. Fig. S15. Effect of TAS3681, enzalutamide, and actinomycin D on AR mRNA expression in LNCaP cells. Fig. S16. TAS3681 downregulates AR‐Vs protein levels at the translational level. Fig. S17. Effect of TAS3681, enzalutamide on AR‐V7 mRNA expression in SAS MDV No.3‐14 cells. Fig. S18. Changes in body weight during TAS3681 treatment in castrated SCID mice implanted with human prostate cancer SAS MDV No.3‐14 cells. Fig. S19. Effect of TAS3681 on serum prostate‐specific antigen (PSA) levels in castrated [file MOL2-18-1980-s001.zip › Supplementary Figure legend.docx]

**Supplementary Fig. S1** Effect of TAS3681, enzalutamide, and bicalutamide on the transcriptional activity of the wild-type AR in VCaP cells. VCaP cells were transfected with pGL4.36 luciferase (containing androgen-dependent murine mammary tumor virus long terminal repeat) and incubated for one day. VCaP cells were treated with various concentrations of TAS3681, enzalutamide, or bicalutamide with 1nM DHT in steroid-depleted medium for one day before luciferase activity measurements. Results are expressed as mean of sextuplicate wells ± SD. AR, androgen receptor.

**Supplementary Fig. S2** Cell proliferation assay of TAS3681 in AR-negative human cancer cells.

A. MIAPaCa-2 cells, B. HCC1806 cells, **C**. SK-OV-3 cells, D. DU145 cells. Results are expressed as the mean of sextuplicate wells ± SD.

**Supplementary Fig. S3** Cell proliferation assay of TAS3681 in the presence of DHT in AR-negative human cancer cells.

A, HCC1806 cells, B, SK-OV-3 cells. Results are expressed as the mean of sextuplicate wells ± SD.

**Supplementary Fig. S4** Effect of TAS3681, enzalutamide, and TOK-001 on AR and GAPDH protein expression in VCaP cells. VCaP cells were treated with test compounds at the indicated concentrations or DMSO for one day. Cell lysates were subjected to immunoblotting for AR and GAPDH to confirm equal loading. The numbers under each blot represent the expression value of AR protein relative to that in DMSO-treated cells. AR, androgen receptor; Enz, enzalutamide; TOK, TOK-001.

**Supplementary Fig. S5** Expression of AR, ERα, GR, PR-A, PR-B, and GAPDH protein in MCF-7 and T-47D cells after treatment with TAS3681.

MCF-7 cells or T-47D cells were treated with test compounds or DMSO at a concentration of 10 μM for one day. Cell lysates were subjected to immunoblotting for each nuclear receptor and GAPDH to confirm equal loading. Enz, enzalutamide; AR, androgen receptor; ERα, estrogen receptor α; PR-A, progesterone receptor A; PR-B, progesterone receptor B; N.D., not detected.

**Supplementary Fig. S6** Effect of antiandrogens on AR subcellular localization in the absence of androgen.
　A, Representative confocal microscopic images of U-2 OS tomato-AR cells treated with 10 μM of TAS3681, enzalutamide, bicalutamide, or 1 nM DHT in steroid-depleted medium. The fluorescence images are cropped from photographs taken at a magnification of 10× using an objective lens and a dichroic mirror. B, U-2 OS tomato-AR cells were treated with 1 nM DHT or 1, 10 µM TAS3681, enzalutamide, and bicalutamide in steroid-depleted medium for 2 h and imaged using an imaging cytometer. The ordinate represents the ratio of fluorescent intensities of nuclear (shown as N) to cytoplasmic (shown as C) as the N/C ratio. Results are expressed as the mean of sextuplicate wells ± SD. **p* < 0.05, ****p* < 0.001 versus DMSO treated cells using Dunnett’s test. Enz, enzalutamide; Bic, bicalutamide.

**Supplementary Fig. S7** Antagonist activities of hydroxyflutamide, bicalutamide, enzalutamide, and TAS3681 against wild-type or mutant ARs; HKE293 cells were transfected with expression vector of wild-type or mutant AR and pGL4.36 luciferase plasmid and incubated for one day. For analysis of the T878A mutant AR, LNCaP cells were transfected with pGLPE luciferase plasmid and incubated for one day. Each cell culture was treated with the indicated compounds with 1 nM DHT in steroid-depleted medium for one day before luciferase activity measurements. Results are expressed as the mean of sextuplicate wells ± SD. ***p* < 0.01 versus DMSO-treated cells using Wilcoxon test, #*p* < 0.05; ##*p* < 0.01; ###*p* < 0.001 versus DHT-treated cells using Dunnett’s test. AR, androgen receptor.

**Supplementary Fig. S8** Effect of TAS3681, enzalutamide, apalutamide, and darolutamide on the transcriptional activity of mutated and wild-type AR. HEK293 cells were transfected with the pGL4.36 plasmids and mutant AR expression vectors and then incubated for one day with DMSO or 0.0003, 0.000949, 0.003, 0.00949, 0.03, 0.0949, 0.3, 0.949, 3, 9.49, 30 µM of TAS3681, enzalutamide, apalutamide, or darolutamide in the presence of DHT. Cells were subsequently harvested, and luciferase activity was determined. Results are expressed as the mean of triplicate wells ± SD. # More than 5% increase of relative luciferase activity (%control) was observed at 30 μM compared with that at 10 μM.

**Supplementary Fig. S9** Downregulation of AR-V7 protein expression in SAS MDV No. 3-14 cells treated with TAS3681. SAS MDV No. 3-14 cells were treated with TAS3681 or enzalutamide for one day at the indicated concentrations. Cell lysates were subjected to immunoblotting for AR-V7 and GAPDH to confirm equal protein loading. Enz, enzalutamide; AR-V7, androgen receptor splice variant 7.

**Supplementary Fig. S10** Downregulation of AR protein expression in SAS MDV No. 3-14 cells treated with TAS3681. SAS MDV No. 3-14 cells were treated with TAS3681 or enzalutamide for one day at the indicated concentrations under DHT containing medium. Cell lysates were subjected to immunoblotting for AR and GAPDH to confirm equal protein loading. Enz, enzalutamide; AR, androgen receptor.

**Supplementary Fig. S11** Effect of TAS3681, enzalutamide, and bicalutamide on AR-FL, AR-Vs, and GAPDH protein expression in 22Rv1 cells; 22Rv1 cells were treated with test compounds at the indicated concentrations or DMSO for one day. Cell lysates were subjected to immunoblotting for AR (N-terminal) and GAPDH to confirm equal loading. The numbers under each blot represent the expression value of AR-FL or AR-Vs protein relative to that in DMSO-treated cells. Enz, enzalutamide; Bic, bicalutamide; AR-FL, androgen receptor full length; AR-Vs, androgen receptor splice variants.

**Supplementary Fig. S12** Effect of TAS3681 on AR-V7-related target genes in SAS MDV No. 3-14 cells. SAS MDV No. 3-14 cells were cultured under androgen-deprived conditions for one day and　then treated with DMSO or 7.5 µM of TAS3681 or enzalutamide in the absence or presence of DHT for one day. Total RNA was extracted from the cells, and cDNA was synthesized. The mRNA expression of *CDK1*, *CDC20*, and *CCNA2* in SAS MDV No. 3-14 cells was analyzed by RT-qPCR analysis. The level of each target gene was normalized to the corresponding expression of *GAPDH*. The relative mRNA expression in DMSO-treated cells without DHT was set as 1. Results are expressed as mean of triplicate samples ± SD. #*p* < 0.05; ##*p* < 0.01; ###*p* < 0.001 versus DMSO treated cells using Dunnett’s test. TAS, TAS3681; Enz, enzalutamide; AR-V7, androgen receptor splice variant 7; CDC20, cell division cycle 20; CDK1, cyclin-dependent kinase 1; CCNA2, cyclin A2.

**Supplementary Fig. S13** Effect of TAS3681 and 17-AAG on the stability of AR and β-actin proteins in LNCaP cells. LNCaP cells were treated with TAS3681 at concentrations of 5 and 10 μM or with 17-AAG at a concentration of 1 μM for 0, 4, 8, or 24 h in the presence of CHX (10 µg/mL). The expression levels of AR and β-actin proteins were evaluated by WB analysis. CHX, cycloheximide.

**Supplementary Fig. S14** Effect of TAS3681 and CHX on AR and GAPDH protein expression in LNCaP cells in the presence of Act D. LNCaP cells were treated with TAS3681 at a concentration of 5 and 10 μM or with CHX at a concentration of 30 µg/mL for 0, 4, 8, or 24 h in the presence of Act D (5 µg/mL). The expression of AR and GAPDH proteins was evaluated by WB analysis. AR, androgen receptor; CHX, cycloheximide; Act D, actinomycin D.

**Supplementary Fig. S15** Effect of TAS3681, enzalutamide, and Act D on *AR* mRNA expression in LNCaP cells. LNCaP cells were cultured under androgen-deprived conditions for one day. LNCaP cells were treated with TAS3681, enzalutamide, or Act D for 4, 8, or 24 hours. The mRNA expression of *AR* and *GAPDH* was evaluated by RT-qPCR analysis. The level of *AR* mRNA was normalized to the corresponding *GAPDH* mRNA level, and relative *AR* mRNA expression in DMSO treated cells was set as 1. Results are expressed as the mean of duplicate samples. AR, androgen receptor; Enz, enzalutamide; Act D, actinomycin D.

**Supplementary Fig. S16** TAS3681 downregulates AR-Vs protein levels at the translational level. A. Effect of TAS3681 on the stability of AR-Vs and GAPDH proteins in SAS MDV No.3-14 cells. SAS MDV No.3-14 cells were treated with TAS3681 at concentrations of 5 and 10 μM for 0, 4, 8, or 24 h in the presence of CHX (3 µg/mL). The expression levels of AR-Vs and GAPDH proteins were evaluated by WB analysis. Results are expressed as mean of triplicate samples ± SD. B. Effect of TAS3681 on the stability of *AR-V7* mRNA in SAS MDV No.3-14 cells. SAS MDV No.3-14 cells were treated with TAS3681 at a concentration of 5 or 10 μM for 0, 4, 8, or 24 h in the presence of Act D (5 μg/mL). The expression of *AR-V7* mRNA was evaluated by RT-PCR. The expression level of *AR* mRNA in the control group (0 h sampling group) was set to 1 to evaluate the relative expression of *AR* mRNA at each sampling time. Results are expressed as mean of triplicate samples ± SD. C. Effect of TAS3681 and CHX on AR-Vs and GAPDH protein expression in SAS MDV No.3-14 cells in the presence of Act D. SAS MDV No.3-14 cells were treated with TAS3681 at a concentration of 5 and 10 μM for 0, 4, 8, or 24 h in the presence of Act D (5 µg/mL). The expression of AR-Vs and GAPDH proteins was evaluated by WB analysis. Results are expressed as mean of triplicate samples ± SD. **p* < 0.05; ***p* < 0.01; ****p* < 0.001 versus DMSO-treated group using Student’s t-test.

AR-Vs, androgen receptor splice variants; CHX, cycloheximide; Act D, actinomycin D.

**Supplementary Fig. S17** Effect of TAS3681, enzalutamide on *AR-V7* mRNA expression in SAS MDV No.3-14 cells. SAS MDV No.3-14 cells were cultured under androgen-deprived conditions for one day. SAS MDV No.3-14 cells were treated with TAS3681 or enzalutamide for 4, 8, or 24 hours. The mRNA expression of *AR-V7* and *GAPDH* was evaluated by RT-qPCR analysis. The level of *AR-V7* mRNA was normalized to the corresponding *GAPDH* mRNA level, and relative *AR-V7* mRNA expression in DMSO treated cells was set as 1. Results are expressed as the mean of triplicate samples ± SD. Enz, enzalutamide; AR-V7, androgen receptor splice variant 7.

**Supplementary Fig. S18** Changes in body weight during TAS3681 treatment in castrated SCID mice implanted with human prostate cancer SAS MDV No.3-14 cells. Mice were orally administrated vehicle or TAS3681 (7.5, 15, or 22.5 mg/kg/day) twice a day for 14 days. Results are expressed as the mean ± SE (n = 10).

**Supplementary Fig. S19** Effect of TAS3681 on serum PSA levels in castrated SCID mice bearing SAS MDV No. 3-14 human prostate cancer xenografts. On days 1 or 15, blood was collected, and serum was prepared. The PSA ratio indicated a fold change from the baseline after 14 days of treatment. Plot shown PSA fold change from baseline PSA levels of individual mice. PSA, prostate-specific antigen.

**Supplementary Fig. S20** Downregulation of AR-FL (left) and AR-Vs protein (right) expression in tumors of castrated SCID mice after treatment with TAS3681. Tumors were harvested on day 15, and tumor tis-sue lysates were subjected to immunoblotting for AR-FL, AR-Vs, and β-actin. The expression of AR-FL protein or AR-Vs protein in each tumor tissue was normalized to that of β-actin, and the relative AR-FL and AR-Vs expression in the vehicle-treated group was set as 1. AR-FL, androgen receptor full length; AR-Vs, androgen receptor splice variants.

**Supplementary Table S1.** qPCR primers

**Supplementary Table S2.** Inhibition of [^3^H]methyltrienolone binding to wild-type AR and T878A mutant AR by TAS3681

**Supplementary Table S3.** Effects of TAS3681 on AR wild type and T878A mutants in cell-based transactivation assays

**Supplementary Table S4.** Effects of TAS3681 on DHT-induced proliferation of VCaP (AR wild type) and LNCaP (T878A mutants)

**Supplementary Table S5.** Inhibition of AR nuclear translocation in U-2 OS tomato-AR cells

**Supplementary Table S6.** IC_50_ values of AR antagonist against mutant and wild-type AR activation by DHT
